# Attention to principles of exercise training: an updated systematic review of randomized controlled trials in cancers other than breast and prostate

**DOI:** 10.1186/s12885-021-08701-y

**Published:** 2021-11-05

**Authors:** Kelcey A. Bland, Sarah E. Neil-Sztramko, Kendra Zadravec, Mary E. Medysky, Jeffrey Kong, Kerri M. Winters-Stone, Kristin L. Campbell

**Affiliations:** 1 Mary MacKillop Institute for Health Research, Australian Catholic University, Melbourne, Victoria Australia; 2 Department of Health Research Methods, Evidence and Impact, McMaster University, Hamilton, Ontario Canada; 3 Rehabilitation Sciences, University of British Columbia, Vancouver, British Columbia Canada; 4 Knight Cancer Institute, Oregon Health & Science University, Portland, OR USA; 5 Department of Physical Therapy, University of British Columbia, 212-2177 Wesbrook Mall, Vancouver, BC V6T 1Z3 Canada

**Keywords:** Neoplasms, Oncology, Exercise prescription, Resistance training, Aerobic exercise

## Abstract

**Background:**

The primary objective of this systematic review was to update our previous review on randomized controlled trials (RCTs) of exercise in cancers other than breast or prostate, evaluating: 1) the application of principles of exercise training within the exercise prescription; 2) reporting of the exercise prescription components (i.e., frequency, intensity, time, and type (FITT)); and 3) reporting of participant adherence to FITT. A secondary objective was to examine whether reporting of these interventions had improved over time.

**Methods:**

MEDLINE, EMBASE, CINAHL and SPORTDiscus databases were searched from 2012 to 2020. Eligible studies were RCTs of at least 4 weeks of aerobic and/or resistance exercise that reported on physiological outcomes relating to exercise (e.g., aerobic capacity, muscular strength) in people with cancer other than breast or prostate.

**Results:**

Eighty-six new studies were identified in the updated search, for a total of 107 studies included in this review. The principle of specificity was applied by 91%, progression by 32%, overload by 46%, initial values by 72%, reversibility by 7% and diminishing returns by 5%. A significant increase in the percentage of studies that appropriately reported initial values (46 to 80%, *p* < 0.001) and progression (15 to 37%, *p* = 0.039) was found for studies published after 2011 compared to older studies. All four FITT prescription components were fully reported in the methods in 58% of all studies, which was higher than the proportion that fully reported adherence to the FITT prescription components in the results (7% of studies). Reporting of the FITT exercise prescription components and FITT adherence did not improve in studies published after 2011 compared to older studies.

**Conclusion:**

Full reporting of exercise prescription and adherence still needs improvement within exercise oncology RCTs. Some aspects of exercise intervention reporting have improved since 2011, including the reporting of the principles of progression and initial values. Enhancing the reporting of exercise prescriptions, particularly FITT adherence, may provide better context for interpreting study results and improve research to practice translation.

**Supplementary Information:**

The online version contains supplementary material available at 10.1186/s12885-021-08701-y.

## Introduction

The number of randomized controlled trials (RCTs) evaluating the role of exercise in an oncology setting has risen sharply. Accumulating evidence suggests exercise can be prescribed as an adjunct therapy to manage the acute and long-term adverse effects of anticancer therapies and improve overall health and survivorship after a cancer diagnosis [1, 2]. While initial research in exercise oncology confirmed these benefits primarily in women with early-stage breast cancer [3–5], followed by men with prostate cancer [6–8], the past decade has seen a surge in RCTs demonstrating similar benefits in numerous other cancer types [2], as well as in advanced cancer and in the palliative care setting [9, 10].

Within the field of exercise oncology, a new appreciation for greater precision in prescribing exercise has emerged, with the aim to optimally target specific patient symptoms and health needs [11–14]. An exercise intervention for individuals with cancer can be prescribed or “dosed” to enhance its efficacy, which can be accomplished through the application of well-established exercise training principles: *specificity, progression, overload, initial values, reversibility* and *diminishing returns* (Table 1)*.* For example, in a study exploring the effect of exercise on aerobic capacity, enrolling participants who report low baseline levels of moderate-to-vigorous physical activity or who are actively undergoing therapies associated with reductions in aerobic capacity would meet the principle of *initial values*, as such participants are more likely to benefit from an aerobic exercise intervention. Once the prescription has been designed based on these principles of training, the delivery of the prescription is operationalized by the appropriate manipulation of the “FITT” exercise formula: *Frequency, Intensity, Time,* and *Type*. Adequate exercise intervention replication or translation into a “real-world” setting requires clinical trials to report these FITT prescription components in the *planned exercise prescription*, along with participant *adherence* to each FITT prescription component. This allows for clarity on the exercise dose prescribed and the actual dose received, which oftentimes can differ based on variability in the tolerance of the study sample to the prescribed program. Failing to consider differences in adherence rates to an exercise intervention could result in drawing false conclusions about efficacy of a specific exercise prescription. Adhering to the principles of exercise training and use of FITT for prescription and adherence recording avoids mistakenly drawing conclusions that are due to poor intervention design, monitoring, or reporting, versus a lack of a true effect.

**Table 1:** Principles of exercise training

Principle	Criteria for this review	Example
**Specificity:** Training adaptations are specific to the organ system or muscles trained with exercise	Appropriate population targeted and modality selected based on primary outcome	Aerobic exercise such as brisk walking is more appropriate for an intervention aimed at increasing cardiovascular fitness than strength training
**Progression:** Over time, the body adapts to exercise. For continued improvement, the volume or intensity of training must be increased	Stated exercise program was progressive and outlined training progression	Increase duration of walking program by 5% every 2 weeks depending on exercise tolerance
**Overload:** For an intervention to improve fitness, the training volume must exceed current habitual physical activity and/or training levels	Rationale provided that program was of sufficient intensity/exercise prescribed relative to baseline capacity	Prescribing intensity in a resistance training program based on % of measured and/or estimated 1-repetition maximum
**Initial values:** Improvements in the outcome of interest will be greatest in those with lower initial values	Selected population with low level of primary outcome measure and/or baseline physical activity levels	Selecting a sample with high baseline fatigue levels to participate in an aerobic training program to increase cardiovascular fitness and reduce fatigue
**Reversibility:** Once a training stimulus is removed, fitness levels will eventually return to baseline	Performed follow-up assessment on participants who decreased or stopped exercise training after conclusion of intervention	Participants who maintained training after a supervised exercise program preserved strength whereas those who stopped exercising returned to baseline
**Diminishing returns:** The expected degree of improvement in fitness decreases as individuals become more fit, thereby increasing the effort required for further improvements. Also known as the ‘ceiling effect’	Performed follow-up assessment of primary outcomes on participants who continued to exercise after conclusion of intervention	Gains in muscle strength are greatest in the first half of a training program unless the training stimulus continually increases

(Campbell et al., 2012 [15]; Winters-Stone et al., 2014 [16]; Neil-Sztramko et al., 2017 [17]; Neil-Sztramko et al., 2019) [18]

Our group first published two systematic reviews in 2012 and 2014 that summarized the utilization of the exercise training principles and associated adherence outcomes in a total of 29 RCTs in women with breast cancer [15] and 33 RCTs in all other cancer types [16]. We concluded that most exercise training principles were inconsistently incorporated within studies and adherence to the FITT prescription components was rarely adequately reported. This conclusion, along with the exponential increase in published exercise oncology RCTs in the past decade, prompted our group to update our previous reviews in breast cancer [17] and prostate cancer [18] to track the field’s progress in this area. Given a major shift in the focus towards evaluating the role of exercise across a diverse range of cancer types and treatments, this updated systematic review summarizes the literature to-date in all other cancer types (namely, cancers other than breast and prostate). In this updated review, our primary aim was to evaluate: 1) the use of the principles of exercise training in the design of the exercise prescription; 2) the reporting of the FITT exercise prescription components in the study methods; and 3) the reporting of participant *adherence* to the FITT prescription components in the study results. A secondary aim was to explore whether any improvement in reporting on the principles of exercise training and FITT exercise prescription components and adherence had occurred since our last publication.

## Methods

This systematic review followed the same protocol reported previously [15–18]. A search of MEDLINE, CINAHL, SPORTDiscus and EMBASE databases was conducted with dates ranging from January 1, 2012 to September 23, 2020, following up on the last search conducted from 1990 to December 31, 2011. Studies that included participants with any cancer diagnosis other than prostate cancer from our previously published review [16] were also included in this review. The search terms, as previously used, included cancer (neoplasm, carcinoma) and exercise (physical activity, aerobic, resistance, walking) specified for each database, in combination with the AND term. Only English-language publications were included. Other relevant systematic reviews were manually searched for relevant publications for inclusion. The protocol was not registered, as our original review [16] commenced prior to the launch of PROSPERO. Because the protocol is already published, we did not register this updated review.

Eligibility criteria included: 1) RCTs with one or more arms involving at least 4 weeks of aerobic and/or resistance exercise; 2) reported one physiological outcome related to exercise (e.g., aerobic capacity, muscular strength, physical function, body composition); and 3) included patients with a cancer diagnosis other than *only* breast or prostate. The criteria of a minimum 4 weeklong exercise intervention and reporting on physiological outcomes relating to exercise were not applied in our original 2014 review [16]. As a result, studies included in our 2014 review that did not meet these updated criteria were excluded from the current analysis. Exclusion criteria included: 1) alternative forms of exercise (e.g., yoga, tai chi) or complimentary alternative methods (i.e., physical therapy, stretching); 2) studies that *only* included patients with metastatic or incurable cancer diagnoses (e.g., inoperable lung cancer); and 3) studies that focused on prehabilitation (i.e., exercise exclusively prior to surgery), or physical activity and/or nutrition behaviour change.

Four reviewers (KB, KZ, MM and SNS) independently determined eligibility using an online software system (Covidence Systematic Review software, Veritas Health Innovation, Melbourne, Australia). Article titles and abstracts were screened for study eligibility and full-text versions of relevant papers were then reviewed to determine eligibility. Discrepancies were discussed and resolved by the input of a senior team member (SNS, KWS, and KC), as required. Reviewers independently extracted relevant data using the online software system (KB, KZ, MM and JK), followed by a discussion and resolution of discrepancies between reviewers or by a third reviewer (SNS, KWS, and KC). Data extraction included: cancer type, sample size, timing of intervention delivery (during or after cancer treatment), treatment type, intervention duration and mode of delivery (supervised or home-based), timing of follow-up measures, primary outcomes, secondary physical fitness and physiological outcomes, and reported study findings. “FITT” (*frequency* of sessions per week, relative or absolute *intensity* of exercise, *time* (duration) of exercise, and *type* of exercise) was used to summarize the exercise prescription. Participant adherence to each FITT prescription component was also extracted where reported by study authors.

For all exercise training principles, reporting of FITT prescription components, and reporting of participant adherence to FITT prescription components, reviewers independently allocated a rating system where a ‘+’ was assigned when the outcome was comprehensibly reported, a ‘NR’ was assigned when the outcome was not reported in the exercise prescription, and a ‘?’ was assigned when the outcome was mentioned but the description was unclear and would not allow for intervention replication. These ratings were also applied to the reporting of participant adherence to the prescription. All available publications and supplementary files were reviewed for a given study to determine ratings. For multi-arm trials, the training principles were considered separately for each intervention arm. For RCTs included in our previous 2014 review across all cancer types [16], if new publications from the same study or data set were identified, ratings were updated in the present review, if more information was available in the newer publication. In line with the methods described in our other updated reviews [17, 18], we report frequencies and percentages of studies meeting the criterion for each training principle, FITT prescription component, and participant adherence to each FITT prescription component.

To determine whether there had been an improvement in the reporting of each exercise training principle, FITT prescription components, and adherence to FITT components since our original search for our previous review [16], a chi-square test was used to calculate the difference in the number of studies reporting ‘+’ versus ‘?’ or ‘NR’ in studies published after 2011 compared to older studies.

## Results

**Fig. 1:**
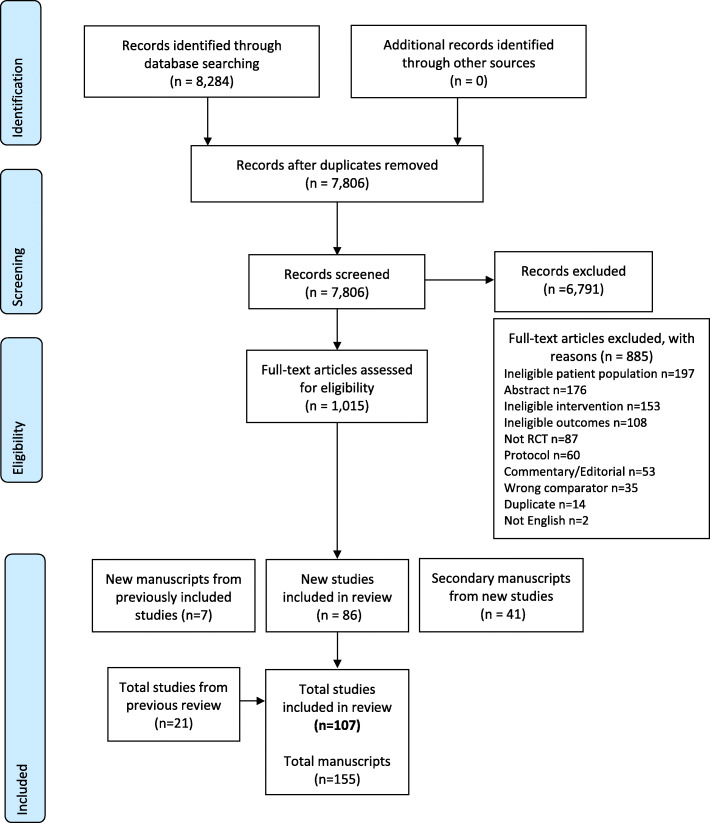
PRISMA flow diagram

The flow chart of search results and study selection process is shown in Fig. 1. A total of 127 new manuscripts, describing 86 studies were identified in our updated search. Of the 33 studies included in the last review, five studies were not included in the current review’s analysis as they did not meet our updated inclusion criteria of a four week minimum intervention [19–22] and reporting on physiological outcomes relating to exercise [23]. Three previous studies [24–26] had a total of seven additional papers published after the original search [27–33]. Upon reviewing newly published manuscripts for previous studies, no changes to the study ratings were made. The results for seven prostate cancer studies from our previous 2014 review were included in a separate updated review specifically in prostate cancer studies [18]. Thus, a total 107 studies, with 122 distinct intervention arms, were included in the final analysis.

A comprehensive description of all evaluated studies, including patient population, sample size, intervention, and outcome measures, is provided in Supplementary File 1. There were 58 (54%) studies conducted among adults diagnosed with solid tumours [24, 34–90], 25 (23%) studies in haematological cancers [25, 91–114] and 24 (22%) studies including patients with mixed cancer diagnoses [26, 115–137]. For the studies in solid tumours, exercise interventions were delivered during cancer treatment in 20 (34%) studies [34–53], during and after treatment in 12 (21%) studies [24, 54–64], and entirely after treatment in 26 (45%) studies [65–90]. The most common solid tumour groups investigated were cancers of colon or rectum (*n* = 15, 26%), lung (*n* = 12, 21%), and head and neck (*n* = 10, 17%). For studies in haematological cancers, 13 (52%) delivered exercise interventions during treatment [91–103], specifically stem-cell transplant or chemotherapy, four (16%) during and after treatment [25, 104–106] and eight (32%) after treatment [107–114]. In studies that enrolled adults with mixed cancer diagnoses, seven (29%) of these studies delivered interventions during cancer treatment [26, 115–120], six (25%) during and after treatment [121–126], and 11 (46%) after treatment [127–137].

### Application of the principles of exercise training

**Fig. 2:**
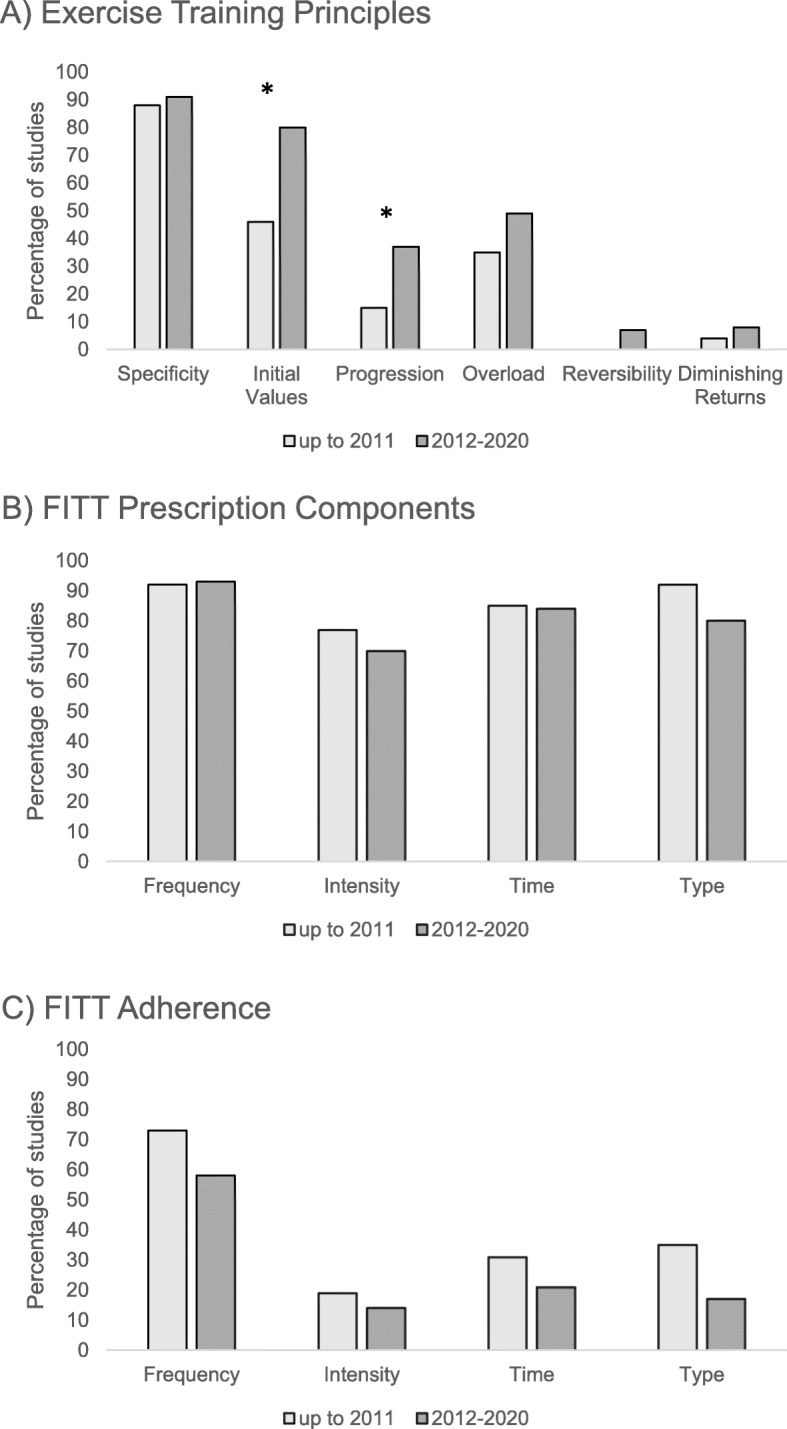
Full Reporting of **A)** Exercise Training Principles, **B)** FITT Prescription Components and **C)** Adherence to FITT Prescription Components. Percentage of studies published between 2012 and 2020 compared to studies published up to 2011 that fully reported ‘+’ each outcome. *Significant difference in the percentage of studies published between 2012 and 2020 compared to studies published up to 2011 that fully reported applying the principles of *initial values* and *progression* (*p* < 0.05). No significant difference in the reporting of FITT prescription components or adherence to FITT prescription components

Ratings for the application of the exercise training principles for all studies and intervention arms, categorized by tumour and treatment type, are shown in Table 2. Differences in the reporting of training principles by study publication year are depicted in Fig. 2A. Only two studies fully reported and applied all six training principles to their interventions. Full application and reporting of at least half of the training principles (i.e., three out of a total six) was found for 53 (49%) studies in total. Five (4%) studies did not adequately report applying any of the six training principles.

**Table 2:** Reporting of the principles of exercise training, FITT prescription components and adherence

	Training principles						Exercise prescription				Adherence				Significant between group differences
Reference	Sp	Pr	Ov	IV	Rev	DR	F	I	T	T	F	I	T	T	
Solid tumours															
*During treatment*															
Arbane 2011 [34]	+	NR	NR	NR	NR	NR	?	?	?	?	NR	NR	NR	NR	↑ Leg strength (inpatient only)
Backman 2014 [35]	+	NR	+	NR	NR	?	+	NR	+	+	+	NR	+	+	None
Capozzi 2016 [36]	+	+	+	+	?	?	+	+	?	?	+	NR	+	NR	None
Christensen 2014 [37]	+	+	+	+	?	?	+	+	+	+	+	NR	NR	NR	None
Grote 2018 [38]	+	+	+	+	NR	NR	+	+	+	+	+	+	?	?	None
Hammer 2020 [39]	+	NR	+	+	NR	NR	+	+	+	+	NR	NR	NR	NR	None
Kamel 2020 [40]	+	+	+	+	NR	NR	+	+	+	+	NR	NR	NR	NR	↑ 6 m walk test, 400 m walk test, chair rise test, isokinetic knee ext./elbow flex/ext., isometric knee ext./elbow flex/ext., LBM ↓ %BF
Lin 2014 [41]	?	?	NR	+	NR	NR	+	?	+	+	+	NR	NR	NR	None
Moller 2015 [42]	+	+	+	+	NR	?	+	+	?	+	+	NR	NR	NR	↑ VO_2_peak
Mustian 2009 [43]	+	?	?	NR	?	+	+	+	+	+	?	NR	+	+	↓ Fatigue*
Rogers 2013 [44]	+	+	NR	+	NR	NR	+	+	+	+	+	NR	NR	?	None
Samuel 2013 [45]	+	?	NR	+	NR	NR	+	+	+	+	NR	NR	NR	NR	↑ 6MWT*
Samuel 2019 [46]	+	NR	NR	+	?	?	+	+	+	+	+	NR	NR	NR	↑ 6MWT*
Sandmael 2017 [47]	+	?	NR	+	?	?	+	?	+	+	+	NR	NR	NR	None
Stuecher 2019 [48]	+	?	?	+	?	?	+	+	+	+	?	?	+	+	↑ SPPB, postural stability, LBM
VanVulpen 2016 [49]	+	+	+	+	?	?	+	+	+	?	+	?	+	NR	↓ Fatigue*
Vigario 2011 [50]	NR	NR	+	+	NR	NR	+	+	+	+	NR	NR	NR	NR	None
Xu 2015 [51]	+	NR	NR	+	NR	NR	+	+	+	+	+	+	+	+	↑ BW*, 6MWT*, HGS
Yen 2019 [52]	+	NR	?	+	NR	NR	+	+	+	?	NR	NR	NR	NR	↑ 6MWT*, BPR/HRR ↓ HR/BP/MAP/RPP/RPE
Zhao 2016 [53]	+	?	+	+	?	?	+	+	+	+	+	NR	NR	NR	↑ Knee ext
*During/after treatment*															
Courneya 2003 [54]	+	NR	?	?	NR	NR	+	+	+	+	NR	?	+	+	None
DeNysschen 2011 [55]	+	?	+	+	?	NR	+	+	+	+	+	+	+	?	None
Donnelly 2011 [24]	+	NR	NR	+	?	?	+	NR	?	?	NR	NR	?	NR	↓ Fatigue*
Edvardsen 2015 [56]	+	?	?	+	NR	NR	+	+	?	+	+	+	NR	NR	↑VO_2_peak*, leg press 1RM, stair climb, 30s sit-to-stand, BMI, total muscle mass, Tlco
Granger 2013 [57]	+	+	+	+	NR	NR	+	+	+	+	+	NR	NR	NR	↑, 6MWT
Hoffman 2017 [58]	+	+	NR	+	NR	NR	+	+	+	+	+	NR	NR	NR	↑ 6MWT
Kaibori 2013 [59]	+	NR	?	?	NR	NR	+	?	+	+	NR	NR	NR	NR	↑ VO_2_peak/AT VO_2_, platelet count test, branched-chain amino acid/tyrosine ratio (high frequency subgroup) ↓ BW, FM, insulin, insulin resistance
Onerup 2020 [60]	+	?	NR	+	?	?	+	+	+	+	+	?	?	+	None
Quist 2018 (EE) [61]	+	?	+	+	?	?	+	+	+	+	NR	NR	NR	NR	↑ VO_2_peak* (26 wks), ↑ 6MWT (14 wks) ↑ FEV1 (14, 26, 52 wks)
Quist 2018 (LE) [61]	+	?	+	+	?	?	+	+	+	+	NR	NR	NR	NR	↑ 6MWT ↑ FEV1 (26 wks)
Salhi 2015 [62]	+	NR	+	+	NR	NR	+	+	+	+	?	NR	NR	NR	↑ 6MWT*
Sommer 2016 (EE) [63]	+	+	+	+	?	?	+	+	+	+	?	?	?	?	None
Sommer 2016 (LE) [63]	+	?	+	?	?	?	+	+	+	+	+	?	NR	?	None
Stigt 2013 [64]	NR	NR	?	+	?	?	+	?	NR	?	+	NR	NR	NR	↑ 6MWT
*After treatment*															
Adams 2017 [65]	+	+	+	+	?	?	+	+	+	+	+	+	NR	?	↑ VO_2_peak *, HRR, respiratory sinus arrhythmia, carotid distensibility, brachial diameter, velocity time integral ↓ HR, DBP, carotid intima-media thickness, carotid-femoral PWV, femoral-toe PWV, CRP, LDL
Arbane 2014 [66]	+	?	+	+	NR	NR	+	+	?	+	NR	NR	NR	NR	↑ Leg strength (subgroup)
Bourke 2011 [67]	+	NR	NR	+	NR	NR	+	+	+	+	+	+	+	+	↑ Aer capacity, 30s sit-to-stand
Brocki 2014 [68]	+	+	+	+	?	?	+	+	+	+	NR	NR	NR	NR	None
Brown 2017 (high dose) [69]	+	+	+	+	NR	NR	?	+	+	+	+	+	+	+	↑ BMD, 6MWT ↓ Visceral adipose tissue, WC, sICAM-1
Brown 2017 (low dose) [69]	+	+	+	+	NR	NR	?	+	+	+	+	+	+	+	↑ BMD, 6MWT ↓ Insulin resistance, sICAM-1
Cavalheri 2017 [70]	+	?	+	+	NR	NR	+	+	+	+	NR	NR	NR	NR	↑VO_2_peak*, 6MWT
Chang 2020 [71]	+	NR	?	+	NR	NR	+	+	+	+	NR	NR	NR	NR	↑ VO_2_peak, 6MWT, albumin
Christensen 2019 [72]	+	?	+	+	?	?	?	+	+	+	?	+	+	+	↓ Glucose AUC, FM ↑ Matsuda index,
Crawford 2017 [73]	?	+	NR	+	NR	NR	+	NR	+	+	+	NR	NR	?	↑ 6MWT, 30s sit-to-stand, arm curl test, HGS, 8 ft. up-and-go, sit-and-reach
Devin 2016 (HIE) [74]	+	NR	+	NR	NR	NR	+	+	+	+	+	+	+	+	↑ VO_2_peak, PPO ↓ BW
Devin 2016 (MIE) [74]	+	NR	+	NR	NR	NR	+	+	+	+	+	+	+	+	None
Devin 2018 (HIIE) [75]	+	NR	+	?	+	?	+	+	+	+	+	+	+	+	↑ VO_2_peak ↓ FM
Devin 2018 (HIIE-T) [75]	+	NR	+	?	+	?	+	+	+	+	+	+	+	+	↑ VO_2_peak
Devin 2018 (MICE) [75]	+	NR	+	?	+	?	+	+	+	+	+	+	+	+	None
Gehring 2018 [76]	+	?	+	+	NR	NR	+	+	NR	+	+	+	+	+	None
Hausmann 2018 [77]	+	NR	NR	NR	?	?	+	?	+	?	NR	NR	NR	NR	↑ VO_2_peak
Lee 2013 [78]	+	?	NR	NR	NR	NR	?	?	?	+	?	?	?	NR	None
Lee 2017 [79]	?	?	NR	?	NR	NR	+	?	?	?	NR	NR	NR	NR	↓ Insulin*, TNF-α, ↑ 30s sit-to-stand, push-up, HGS
Lee 2018 [80]	?	NR	NR	?	NR	NR	+	NR	?	?	?	NR	NR	NR	↑ PA levels*, step test, push-up test
Lønbro 2013 (EE) [81]	+	?	NR	NR	?	NR	+	+	+	+	+	NR	NR	NR	↑ LBM, isometric knee ext., isokinetic knee flex (wk 12)
Lønbro 2013 (DE) [81]	+	?	NR	NR	?	NR	+	+	+	+	+	NR	NR	NR	↑ LBM, isometric knee ext./flex, isokinetic knee ext./flex, sit-to-stand, arm curl (wk 24)
Martin 2015 (HIG) [82]	+	?	+	NR	+	?	+	+	+	?	+	?	NR	NR	↑ VO_2_peak
Martin 2015 (LIG) [82]	+	?	+	NR	+	?	+	+	+	?	+	?	NR	NR	↑ VO_2_peak
Mascherini 2020 [83]	+	?	+	+	NR	?	+	?	+	?	?	NR	?	?	↑ 6MWT, 30s sit-to-stand, sit and reach ↓ BW, BMI, HC
McNeely 2008 [84]	+	+	+	+	NR	NR	+	+	+	+	+	NR	NR	NR	↓ Pain & disability* ↑ Chest press/seated row 1RM
Messaggi-Sartor 2019 [85]	+	+	+	+	NR	NR	+	+	+	+	+	NR	NR	+	↑ VO_2_ peak ↑ peak ventilation, max inspiratory pressure, max expiratory pressure, IGFBP-3
Meyerhardt 2020 [86]	+	+	?	+	NR	NR	?	?	+	+	?	?	?	?	↓ Insulin*, hs-CRP, IL6, insulin resistance, BW, BMI, WC
Nuri 2016 [87]	+	NR	NR	NR	+	?	+	?	+	?	NR	NR	NR	NR	↑ Ghrelin*, estimated VO_2_peak ↓ %BF
Pinto 2013 [88]	+	+	+	+	?	?	+	+	+	+	?	NR	+	NR	↑ PA levels*, estimated VO_2_peak*
Porserud 2014 [89]	+	NR	NR	NR	?	?	+	NR	+	?	+	NR	?	NR	↑ 6MWT*
Rossi 2016 [90]	+	?	?	+	NR	NR	+	+	+	+	+	NR	?	?	↑ 6MWT ↓ WC
Haematological cancer															
*During treatment*															
Alibhai 2015 [91]	+	+	+	+	?	?	+	?	+	+	+	NR	+	NR	↑ 6MWT, HGS, 10-chair stand test
Baumann 2010 [92]	+	NR	+	+	NR	NR	+	+	+	+	+	NR	NR	NR	↑ Aer capacity (W, min)*, knee ext.*, QoL*, IVC, FVC
Baumann 2011 [93]	+	NR	?	NR	NR	NR	+	+	+	+	+	NR	NR	NR	↑ Aer capacity (W/kg)*
Bryant 2018 [94]	+	+	?	+	NR	NR	+	+	+	+	+	NR	NR	NR	None
Coleman 2003 [95]	+	NR	?	NR	NR	NR	NR	NR	NR	+	+	NR	NR	NR	↑ LBM
Coleman 2012 [96]	+	NR	NR	+	NR	NR	?	+	+	+	?	NR	NR	NR	None
Duregon 2019 [97]	+	?	?	+	NR	NR	+	+	+	+	NR	NR	NR	NR	None
Jarden 2009 [98]	+	?	?	NR	?	NR	+	?	+	+	+	NR	NR	+	↑ Chest press/leg ext. 1RM, isometric knee ext
Larsen 2019 [99]	+	?	?	+	?	?	+	?	+	+	+	?	?	?	None
Oechsle 2014 [100]	+	NR	?	+	NR	NR	+	?	+	+	+	NR	NR	NR	↑ estimated VO_2_, VE
Santa-Mina 2020 [101]	+	?	+	+	?	?	+	?	+	+	?	?	?	?	None
Streckmann 2014 [102]	+	NR	?	+	?	?	+	+	+	+	?	NR	NR	?	↑ QoL*, peripheral deep sensitivity, balance control on static/dynamic surface & with perturbation
Wehrle 2019 (AER) [103]	+	NR	+	+	NR	NR	+	+	+	+	+	NR	NR	NR	None
Wehrle 2019 (RET) [103]	+	NR	NR	+	NR	NR	+	+	?	+	+	NR	NR	NR	↑ Knee ext./flex
*During/after treatment*															
Courneya 2009 [25]	+	+	+	NR	?	?	+	+	+	+	+	+	+	+	↑ QoL*, VO_2_peak, LBM ↓ %BF
Koutoukidis 2020 [104]	+	+	+	+	?	?	+	?	?	?	?	NR	?	?	↑ Leg ext
Mello 2003 [105]	?	?	NR	NR	NR	NR	+	+	+	+	NR	NR	NR	NR	↑ Hip flex*
Wiskemann 2011 [106]	+	?	+	+	?	?	+	+	+	+	+	NR	NR	NR	↑ 6MWT, lower body strength ↓ Total mortality (after discharge)
*After treatment*															
Alibhai 2014 [107]	+	NR	NR	+	?	?	+	?	+	+	+	NR	?	NR	None
Furzer 2016 [108]	+	+	+	NR	+	+	+	+	+	?	+	+	+	NR	↓ Fatigue* ↑ Aer capacity (W/kg), chest/arms/legs/total strength 1RM, %BF, LBM, BMD
Hacker 2011 [109]	+	?	?	+	NR	NR	+	+	+	+	+	NR	NR	NR	None
Hacker 2017 [110]	+	+	?	+	NR	NR	+	+	NR	+	+	NR	NR	NR	↑ Timed stair climb, TUG
Jarden 2013 [111]	+	?	?	+	NR	NR	+	+	+	+	+	NR	NR	NR	↑ 6MWT*, estimated VO_2_peak, 30s sit-to-stand, arm curl test
Knols 2011 [112]	+	?	?	?	?	?	+	+	+	+	+	NR	NR	NR	↑ 6MWT*, knee ext.*
Persoon 2017 [113]	+	+	+	+	NR	NR	+	+	+	+	+	NR	NR	NR	None
Shelton 2009 (Sup) [114]	+	?	NR	+	NR	NR	+	?	+	+	+	NR	NR	NR	None
Shelton 2009 (HB) [114]	+	?	NR	+	NR	NR	+	?	?	+	NR	NR	NR	NR	None
Mixed cancer types															
*During treatment*															
Adamsen 2009 [26]	+	?	+	NR	NR	NR	+	+	+	+	+	NR	NR	NR	↓ Fatigue* ↑ estimated VO_2_peak, leg press/chest press/pull down 1RM
Arrieta 2019 [115]	+	?	NR	+	?	?	?	?	?	?	?	?	?	?	↑ SPPB* (breast cancer, female, normal nutritional status subgroups only)
Griffith 2009 [116]	+	NR	?	+	NR	NR	+	+	+	+	+	NR	+	+	↑ VO_2_peak (prostate vs non-prostate)
Marechal 2019 [117]	+	?	?	+	NR	NR	+	+	+	+	NR	NR	NR	NR	↑ Sit-to-stand, global physical capacity score
Peterson 2018 [118]	+	+	?	+	NR	NR	+	+	+	+	NR	NR	NR	NR	None
Sturm 2014 [119]	?	NR	NR	+	NR	NR	+	NR	+	+	+	NR	NR	+	↑ 6MWT ↓ Fatigue*
Wenzel 2013 [120]	+	NR	+	+	NR	NR	+	+	+	+	NR	NR	NR	NR	↓ Sleep quality* ↑ Vigour
*During/after treatment*															
Courneya 2003 [121]	+	NR	?	NR	NR	NR	+	+	+	+	NR	?	+	NR	↑ QoL* ↓ %BF
Courneya 2008 [122]	+	NR	+	NR	NR	NR	+	+	NR	+	+	+	+	+	↑ VO_2_peak, PPO, VT
Irwin 2017 [123]	?	?	NR	NR	NR	NR	+	NR	?	?	+	NR	?	NR	↑ 6MWT
Mayo 2014 [124]	+	+	+	?	?	?	+	NR	+	+	+	NR	+	+	None
Schuler 2017 (Sup + HB) [125]	+	NR	NR	+	?	?	+	+	+	?	NR	NR	NR	NR	None
Schuler 2017 (HB) [125]	+	NR	NR	+	?	?	+	+	+	?	NR	NR	NR	NR	None
Schwartz 2009 (AER) [126]	+	NR	NR	+	NR	+	+	NR	+	+	+	NR	NR	NR	↓ Weight gain*, %BF* ↑ 12MWT, overhead press/seated row/leg press 1RM
Schwartz 2009 (RET) [126]	+	?	+	+	NR	+	+	NR	+	+	+	NR	NR	NR	None
*After treatment*															
Broderick 2013 [127]	+	+	+	+	+	+	+	+	+	+	+	+	+	NR	None
Burnham 2002 [128]	+	+	+	NR	NR	NR	+	+	+	+	+	NR	NR	NR	↑ VO_2_peak, flexibility ↓ %BF
Jones 2014 [129]	+	+	+	+	+	+	+	+	+	+	?	NR	+	+	↑ Cardiovascular mortality/hospitalization
Kampshoff 2015 (HI) [130]	+	+	+	?	NR	NR	+	+	+	+	+	?	+	+	↑ VO_2_peak *, PPO*, VT* (HI/LMI) ↓ Fatigue*
Kampshoff 2015 (LMI) [130]	+	+	+	?	NR	NR	+	+	+	+	+	?	+	+	None
Kneis 2019 (AER) [131]	+	?	+	+	NR	NR	+	+	+	+	+	NR	NR	NR	↑ Jump height/P_max_jump,_ vibration sense
Kneis 2019 (AER + balance) [131]	+	?	+	+	NR	NR	+	+	+	+	+	NR	NR	NR	↑ MS_EOunstable_ duration, patella vibration ↓ ST_EO_
Knobf 2017 [132]	+	?	+	+	NR	NR	+	+	+	+	+	NR	?	NR	↑ Aer capacity, HRR ↔ Insulin
LaStayo 2011 [133]	+	+	NR	+	NR	NR	+	+	+	+	+	+	NR	+	↑ Muscle CSA, 6MWT, stair descent
Midtgaard 2013 [134]	+	+	+	+	NR	NR	+	+	+	+	+	?	NR	NR	↑ PA levels*, VO_2_peak*, leg press/chest press 1RM
Pisu 2017 [135]	+	NR	NR	+	NR	NR	+	+	+	+	+	NR	NR	NR	None
Thorsen 2005 [136]	?	NR	?	NR	NR	NR	+	+	+	+	+	NR	NR	+	↑ VO_2_peak*
Toohey 2016 (LVHIIT) [137]	+	+	+	+	NR	NR	+	+	+	+	?	?	?	+	↑ 6MWT
Toohey 2016 (CLMIT) [137]	+	NR	+	+	NR	NR	+	+	+	+	?	?	?	+	None

*Primary outcome where specifically stated, +: clear reporting, *NR:* not reported, ?: unclear reporting, *%BF* body fat %, *1RM* 1-repetition maximum, *12MWT* 12-min walk test, *6MWT* 6-min walk test, *AER* aerobic exercise, *AT* anaerobic threshold, *AUC* area under the curve, *BM* body mass, *BMD* bone mineral density, *BP* blood pressure, *BW* body weight, *CG* control group, *CRP* C-reactive protein, *CSA* cross-sectional area, *DE* delayed exercise, *DR* diminishing returns, *EE* early exercise, *F* frequency, *FM* fat mass, *FVC* forced vital capacity, *HC* hip circumference, *hs-CRP* high-sensitivity C-reactive protein, *HGS* handgrip strength, *HI* high intensity exercise, *HIE* high-intensity exercise, *HIG* high-intensity group, *HIIE-T* high-intensity interval exercise-tapered, *HR* heart rate, *HRR* heart rate recovery, *I* intensity, *IG* intervention group, *IGFBP-3* insulin-like growth factor binding protein-3, *IL6* interleukin 6, *IV* initial values, *IVC* inspiratory vital capacity, *LBM* lean body mass, *LDL* low-density lipoprotein, *LIG* low-intensity group, *LMI* low-to-moderate intensity exercise, *LVHIIT* low-volume high intensity interval training, *MAP* mean arterial pressure, *MICE* moderate intensity continuous exercise, *MIPT* maximum isokinetic peak torque, *MSEO* monopedal stance on stable surface, *MSEOunstable* monopedal stance on unstable surface, *MVIC* maximum voluntary isometric contraction, *Ov* overload, *PA* physical activity, *Pmax_jump* maximum jump power output, *PPO* peak power output, *Pr* progression, *PWV* pulse wave velocity, *QoL* quality of life, *RET* resistance training, *Rev* reversibility, *ROM* range of motion, *RPP* rate pressure product, *sICAM-*1 soluble intercellular adhesion molecule-1, *Sp* specifity, *SPPB* short physical performance battery, *STEO* semi-tandem stance with eyes open, *Sup* supervised, *T* time or type, *TC* total cholesterol, *TG* triglycerides, *Tlco* carbon monoxide transfer factor, *TNF*-α tumour necrosis factor alpha, *TUG* timed up and go, *VE* ventilatory equivalent, *VO*_*2*_ oxygen consumption, *VO*_*2*_*peak* peak oxygen consumption, *VT* ventilatory threshold, *W* watts, *WC* waist circumference

*Specificity* was the most frequently applied training principle and appropriately reported in 97 (91%) studies. Given *specificity* was commonly adequately applied across all studies, no significant difference in the reporting of *specificity* was found among new studies published after 2011 compared to older studies (91% vs. 88%; *p* = 0.659). The principle of *initial values* was the second most applied training principle and was appropriately reported in 77 (72%) studies overall. A significant improvement in the reporting of *initial values* was found for new studies published after 2011 compared to older studies (80% vs. 46%, *p* < 0.001). *Progression* was appropriately reported and applied in 34 (32%) studies. Like *initial values,* appropriate reporting of *progression* also improved in new studies published after 2011 compared to older studies (37% vs. 15%, *p* = 0.039). The principle of *overload* was appropriately applied and reported in 49 (46%) studies in total. While the number of studies adequately reporting *overload* increased in new studies compared to older ones, this difference was not statistically significant (49% vs. 35%, *p* = 0.189). Most studies did not adequately report or unclearly reported the principles of *reversibility* and *diminishing returns. Reversibility* was only appropriately applied in 7 (7%) studies overall, all of which were new studies published after 2011. Reporting of reversibility was not significantly different between new and older studies (7% vs. 0%, *p* = 0.121). *Diminishing returns* was appropriately reported in 5 (5%) studies overall and no difference in the reporting of *diminishing returns* was found between new and older studies (4% vs. 8%, *p* = 0.402).

### Reporting of the FITT prescription components

Ratings for the reporting of the FITT exercise prescription components for all studies and intervention arms are shown in Table 2. Differences in the reporting of the FITT prescription components between new studies published after 2011 compared to older studies is depicted in Fig. 2B. All four FITT prescription components were reported in full within the study methods in 62 out of 107 (58%) studies. There were 97 (91%) studies that fully reported at least half of the FITT prescription components (i.e., at least two out of four). Only two (2%) studies did not fully report any of the four FITT prescription components in the study methods.

The prescribed exercise session *frequency* (i.e., days per week) was the most frequently fully reported FITT prescription component and was reported in 99 (93%) studies overall. There was no difference in the reporting of *frequency* between new studies published after 2011 compared to older studies (93% vs. 92%, *p* = 0.962). The prescribed target exercise *intensity* was fully reported in 77 (72%) studies, with no difference in reporting between new studies published after 2011 and older studies (70% vs. 77%, *p* = 0.518). A target exercise duration, or *time,* was fully reported for 90 (84%) studies overall and no difference in the reporting of the prescribed exercise *time* was found between new studies and older studies (85% vs. 85%, *p* = 0.936). Lastly, the prescribed exercise *type* was fully reported for 89 (83%) studies overall. Similarly, there was no difference in the reporting of exercise *type* between new studies and older studies (80% vs. 92%, *p* = 0.153).

### Reporting of FITT prescription adherence

Ratings for the reporting of participant *adherence* to the FITT exercise prescription components for all studies and intervention arms are shown in Table 2. Full reporting of adherence to the FITT prescription components within the study results is shown for new studies published after 2011 compared to older studies in Fig. 2C. In total, adherence to all four FITT prescription components was reported in the study results for 8 out of 107 (7%) total studies. Adherence to at least half of the FITT prescription components was reported in 31 (29%) studies. There were 33 (31%) studies that did not fully report adherence to *any* FITT prescription component. All evaluated studies appeared to be much more likely to fully report all four FITT prescription components in the study methods (58%) compared to *adherence* to all four FITT prescription components in the study results (7%).

Adherence to exercise session *frequency* (i.e., attendance) was the most fully reported adherence outcome and was reported in 66 (62%) studies overall. Among new studies published after 2011, only 58% of studies fully reported adherence to exercise session *frequency* compared to 73% of older studies (*p* = 0.170). There was full reporting of exercise adherence to *intensity* for 16 (15%) studies in total, with no difference between new studies and older studies (14% vs. 19%, *p* = 0.482). Adherence to exercise *time* was fully reported for 25 (23%) studies overall*,* with no difference between new studies and older studies (21% vs. 31%, *p* = 0.305). Adherence to exercise *type* was fully reported in 23 (22%) studies and there was no difference between new studies and older studies (17% vs. 35%, *p* = 0.061).

## Discussion

In this systematic review, which included data from participants diagnosed with solid tumours and haematological cancers, the overall application and reporting of the principles of exercise training, the FITT prescription components, and exercise adherence varied. Less than 50% of all evaluated studies applied at least half of the exercise training principles (i.e., three or more out of six). However, a significant improvement in the application and reporting of *initial values* (46 to 80%) and *progression* (15 to 37%) was found for studies published after 2011 compared to older studies. Regarding the FITT exercise prescription components, 58% of all evaluated studies reported applying all four FITT prescription components in the study methods, yet a much smaller proportion (7% of studies) reported *adherence* to all four FITT prescription components in the study results. No significant improvements over time were observed in the reporting of the FITT exercise prescription components or adherence to the FITT prescription components.

In this review, *specificity* was the most consistently applied exercise training principle. Specificity requires selecting the exercise modality based on the primary outcome (i.e., to improve aerobic fitness, prescribe aerobic exercise, such as brisk walking). This application should continue to be strong in future trials. To apply the principle of specificity one step further, future research could strive to narrow in on the most appropriate modality of exercise to elicit a training effect. For example, brisk walking may help to improve aerobic fitness in some patients, but other aerobic exercise modalities might be more effective. The elliptical trainer, on the other hand, may allow participants to achieve higher exercise intensities, as it recruits both upper and lower body muscle groups. Almost 70% of all interventions appropriately reported *initial values* and reporting of this principle improved significantly among studies published after 2011. The principle of *initial values* considers participants’ baseline levels of the target outcome of interest (e.g., physical fitness levels), as improvements in the outcome of interest will be greatest in those with lower initial values. Adequate reporting of the principle of *progression* also improved among new studies (22% increase). However, over two-thirds of all studies did not report *progression* or provided an unclear description of *how* exercise was progressed, so that interventions may be replicated (e.g., 5–10% increase in heart rate maximum every 2 weeks for aerobic exercise). The principle of *overload*, which requires exercise to be prescribed based on baseline exercise testing, was less commonly applied, and did not improve in new studies. While studies may be limited by resources, funding, and personnel to conduct gold-standard assessments of physical fitness (e.g., cardiopulmonary exercise testing), submaximal exercise testing protocols or clinical measures of physical function can still be utilized to facilitate prescribing more appropriate exercise targets to participants. Consideration of *reversibility* and *diminishing returns* were the least frequently applied training principles. This is understandable given that a follow up fitness test after completing the intervention may not be done or reported in the primary manuscript. However, performing repeat testing in people who continue to exercise (*diminishing returns*) and people who discontinue exercise (*reversibility*) upon intervention completion can help underscore the importance of delivering an adequate exercise stimulus for continued improvement as well as identify the minimal effective exercise dose required to achieve and maintain exercise health benefits.

The FITT exercise prescription components (i.e., *prescribed* exercise) were consistently more fully reported than participant adherence to the FITT prescription components (i.e., *completed* exercise) among our evaluated studies. Regarding adherence outcomes, *frequency* was the most reported, often as attendance or number of exercise sessions completed. However, we cannot emphasize enough the importance of reporting adherence beyond exercise session attendance; especially in feasibility studies and studies that are delivering ‘novel’ exercise interventions, such as non-linear or high intensity exercise prescriptions, or focusing on understudied cancer populations. Reporting of adherence to exercise intensity and duration, or resistance training volume, can be challenging within studies. However, there are recent publications that are examples of exemplary exercise adherence reporting in oncology and can help guide future research [13, 14, 138–140]. These papers illustrate widespread variations or disruptions in exercise session attendance and prescription adherence among participants and throughout cancer treatment. For example, exercise session attendance and adherence to aerobic exercise was shown to gradually decrease over the course of chemotherapy for breast cancer and over a third of participants required aerobic exercise intensity adjustments due to treatment symptoms [13]. If this study did not report on adherence to the prescribed intensity of exercise, any practical application of this study in clinical settings could risk injuring patients or participant dropout. This data highlights the the necessity of adjusting to participant needs; especially during cancer treatment, when individuals present with cyclic changes in symptom severity between chemotherapy cycles and potentially, accumulating symptom severity as treatment duration lengthens. Less than perfect exercise adherence does not indicate study failure and full reporting of exercise prescription adherence should be considered a strength across exercise oncology RCTs. Adherence reporting and transparency is necessary for continued improvement in exercise intervention design as well as the development of evidence-informed approaches to modifying exercise dose for appropriate translation of RCT findings into clinic and community settings.

For some outcomes, we noted that the reporting of FITT prescription components and adherence trended towards being worse across new studies published after 2011 compared to earlier studies. Full reporting of the prescribed exercise *type*, for example, was seen in 80% of new studies published after 2011 compared to 92% of earlier studies. There are several possible explanations for this observation. Applying the FITT prescription components and reporting adherence is more complex for interventions that prescribe exercise outside of ‘typical’ aerobic or resistance-based training. One example is the study by Crawford et al. that prescribed a wall-climbing intervention to women who had undergone treatment for gynaecological cancer [69]. For this type of intervention, rating of perceived exertion (RPE) [141] and exercise minutes can still be collected as proxies for exercise intensity and duration. For home-based exercise interventions, specifically, many studies prescribed exercise duration as total minutes per week (e.g., 150 min per week of moderate to vigorous exercise) and then reported the total mean minutes per week completed for the intervention group. In addition to this, reporting how long the participants’ mean duration was for a *single* bout of exercise would still be of value (adherence to exercise *time)* as well as the number of days participants chose to exercise per week (adherence to exercise *frequency)*. Moreover, studies prescribing home-based exercise also frequently do not report the prescribed exercise *type,* as it may be self-selected by participants. Reporting some examples of “suggested” exercise types within the study methods, such as walking outside, would clarify the exercise prescription. Reporting the types of exercises participants self-select in the adherence section of results would then provide insight on participant exercise preferences and help inform achievable exercise recommendations in a clinical or community setting. Reporting exercise adherence to resistance training exercise, is also somewhat of a challenge, given there are several variables of interest (i.e., sets, repetitions, weight). For resistance exercise training, prescribing a target RPE and collecting patient-reported RPE following each exercise is a pragmatic approach to exercise prescription and adherence monitoring [142]. RPE helps gauge resistance training intensity and whether patients are achieving volitional fatigue. Training loads can then be adjusted or progressed accordingly to ensure an adequate training stimulus is being applied. Moreover, Fairman et al. have recently provided guidance on how to clearly report adherence to resistance training in exercise oncology [138].

Outside of the exercise oncology literature, it has been shown that completeness or adequacy of intervention reporting is lower in non-pharmacological trials versus pharmacological trials [143]. For all clinical trials, full reporting of a given intervention is essential and goes beyond naming the intervention and listing its main components. There are crucial features of an intervention, including its setting, duration, mode of delivery, monitoring and so forth, which must be adequately described to allow for full intervention interpretation, replication, and implementation. Completeness reporting of exercise oncology trials according to TIDieR (template for intervention description and replication) checklist, for example, has been calculated as ranging from 46 to 96% [144]. The TIDieR checklist contains 12 items: name, why, what (materials), what (procedure), who provided, how, where, when and how much, tailoring, modifications, how well (planned), how well (actual) [145]. We argue that in addition to standard completeness reporting for clinical interventions, as outlined in tools such as the TIDieR checklist, reporting of the exercise training principles and adherence, as discussed in this review, is necessary in exercise oncology for intervention replication and translation. To adopt this approach to study reporting, following the Consensus on Exercise Reporting Template (CERT) guidelines is recommended [146]. CERT highlights how to report frequency, intensity, duration and type of exercise outlined in section 13, “When, How Much” and exercise completed in section 16, “How Well: Planned, actual”.

### Limitations

We did not contact authors for missing information. Interventions may have been designed in line with the principles of exercise training, yet were perhaps not reported this way. Strict journal page limits can be a barrier to full reporting of exercise interventions. Online appendices and supplementary materials, however, can be used to report this information. Further, we did not include single-arm studies, studies in patients with incurable cancer or receiving palliative care, or studies with prehabilitation interventions, alternative exercise (e.g., yoga), physical therapy interventions (e.g., arm rehabilitation and mobilization following breast cancer surgery), or interventions less than 4 weeks. Evaluating the exercise intervention design and adherence for these special cases is likely still valuable and should be considered in future reviews. However, a recent review by Medysky et al. summarizes the reporting of exercise training principles in RCTs of lung cancer and includes both prehabilitation interventions and patients with incurable disease [147]. While we evaluated the application and reporting of exercise training principles, understanding how the application of these principles directly influences fitness and cancer-specific outcomes is an important area for ongoing research. A previous review evaluated how prescribed FITT factors moderated change in physical fitness in those living with and beyond cancer and found that greater exercise frequency and longer session duration resulted in larger effects [148]. Expanding this to include adherence to FITT and the application of the principles of exercise training will help pinpoint which intervention components should receive the greatest consideration in oncology settings.

## Conclusion

With the growing number of exercise oncology trials conducted in a variety of cancer populations every year, appropriate application of the basic principles of exercise training highlighted in the current review is strongly encouraged. Since our previous reviews on this topic, we found that most exercise training principles are still inconsistently reported. However, we did find a significant improvement in the reporting of the principles of *initial values* and *progression* among studies published after 2011. Findings from the current review suggest the reporting of exercise intervention adherence to all four FITT prescription components requires the greatest improvement. The goal of adopting this style of intervention reporting is to facilitate translation into clinical practice, while also ensuring interventions are appropriately designed and monitored to maximize efficacy.

## Supplementary Information

**Additional file 1: Table S1.** Description of studies.

## Data Availability

The datasets used and/or analyzed during the current study are available from the corresponding author on reasonable request.
