# Correction to: Attention to principles of exercise training: an updated systematic review of randomized controlled trials in cancers other than breast and prostate

**DOI:** 10.1186/s12885-021-09022-w

**Published:** 2022-02-17

**Authors:** Kelcey A. Bland, Sarah E. Neil-Sztramko, Kendra Zadravec, Mary E. Medysky, Jeffrey Kong, Kerri M. Winters-Stone, Kristin L. Campbell

**Affiliations:** 1 Mary MacKillop Institute for Health Research, Australian Catholic University, Melbourne, Victoria Australia; 2 Department of Health Research Methods, Evidence and Impact, McMaster University, Hamilton, Ontario Canada; 3 Rehabilitation Sciences, University of British Columbia, Vancouver, British Columbia Canada; 4 Knight Cancer Institute, Oregon Health & Science University, Portland, OR USA; 5 Department of Physical Therapy, University of British Columbia, 212-2177 Wesbrook Mall, Vancouver, BC V6T 1Z3 Canada

**Correction to: BMC Cancer 21, 1179 (2021)**

**https://doi.org/10.1186/s12885-021-08701-y**

Following publication of the original article [1], the authors identified the following typesetting errors:

1. A small number of references were not inserted in-text correctly. As a result, some in-text citations are not correct for specific statements in our results section on pages 4-5. The corrected version should read as follows:
  “There were 58 (54%) studies conducted among adults diagnosed with solid tumours [24, 34-90], 25 (23%) studies in haematological cancers [25, 91-114] and 24 (22%) studies including patients with mixed cancer diagnoses [26, 115-137]. For the studies in solid tumours, exercise interventions were delivered during cancer treatment in 20 (34%) studies [34-53], during and after treatment in 12 (21%) studies [24, 54-64], and entirely after treatment in 26 (45%) studies [65-90]. The most common solid tumour groups investigated were cancers of the colon or rectum (n = 15, 26%), lung (n = 12, 21%), and head and neck (n = 10, 17%). For studies in haematological cancers, 13 (52%) delivered exercise interventions during treatment [91-103], specifically stem-cell transplant or chemotherapy, four (16%) during and after treatment [25, 104-106], and eight (32%) after treatment [107-114]. In studies that enrolled adults with mixed cancer diagnoses, seven (29%) of these studies delivered interventions during cancer treatment [26, 115-120], six (25%) during and after treatment [121-126], and 11 (46%) after treatment [127-137].”
  This in-text error affects downstream citation numbering, including citations in Table 2. It also influences the reference list order.
2. One of the studies in Table 2 appears in the wrong location. The study by Grote et al. should appear under the section “Solid tumours, during treatment.”

Please see below for the corrected Table 2. The reference list has been corrected in the updated article [1].

**Table 2:** Reporting of the principles of exercise training, FITT prescription components and adherence

	Training principles						Exercise prescription				Adherence				Significant between group differences
Reference	Sp	Pr	Ov	IV	Rev	DR	F	I	T	T	F	I	T	T	
Solid tumours															
*During treatment*															
Arbane 2011 [34]	+	NR	NR	NR	NR	NR	?	?	?	?	NR	NR	NR	NR	↑ Leg strength (inpatient only)
Backman 2014 [35]	+	NR	+	NR	NR	?	+	NR	+	+	+	NR	+	+	None
Capozzi 2016 [36]	+	+	+	+	?	?	+	+	?	?	+	NR	+	NR	None
Christensen 2014 [37]	+	+	+	+	?	?	+	+	+	+	+	NR	NR	NR	None
Grote 2018 [38]	+	+	+	+	NR	NR	+	+	+	+	+	+	?	?	None
Hammer 2020 [39]	+	NR	+	+	NR	NR	+	+	+	+	NR	NR	NR	NR	None
Kamel 2020 [40]	+	+	+	+	NR	NR	+	+	+	+	NR	NR	NR	NR	↑ 6 m walk test, 400 m walk test, chair rise test, isokinetic knee ext./elbow flex/ext., isometric knee ext./elbow flex/ext., LBM ↓ %BF
Lin 2014 [41]	?	?	NR	+	NR	NR	+	?	+	+	+	NR	NR	NR	None
Moller 2015 [42]	+	+	+	+	NR	?	+	+	?	+	+	NR	NR	NR	↑ VO_2_peak
Mustian 2009 [43]	+	?	?	NR	?	+	+	+	+	+	?	NR	+	+	↓ Fatigue*
Rogers 2013 [44]	+	+	NR	+	NR	NR	+	+	+	+	+	NR	NR	?	None
Samuel 2013 [45]	+	?	NR	+	NR	NR	+	+	+	+	NR	NR	NR	NR	↑ 6MWT*
Samuel 2019 [46]	+	NR	NR	+	?	?	+	+	+	+	+	NR	NR	NR	↑ 6MWT*
Sandmael 2017 [47]	+	?	NR	+	?	?	+	?	+	+	+	NR	NR	NR	None
Stuecher 2019 [48]	+	?	?	+	?	?	+	+	+	+	?	?	+	+	↑ SPPB, postural stability, LBM
VanVulpen 2016 [49]	+	+	+	+	?	?	+	+	+	?	+	?	+	NR	↓ Fatigue*
Vigario 2011 [50]	NR	NR	+	+	NR	NR	+	+	+	+	NR	NR	NR	NR	None
Xu 2015 [51]	+	NR	NR	+	NR	NR	+	+	+	+	+	+	+	+	↑ BW*, 6MWT*, HGS
Yen 2019 [52]	+	NR	?	+	NR	NR	+	+	+	?	NR	NR	NR	NR	↑ 6MWT*, BPR/HRR ↓ HR/BP/MAP/RPP/RPE
Zhao 2016 [53]	+	?	+	+	?	?	+	+	+	+	+	NR	NR	NR	↑ Knee ext
*During/after treatment*															
Courneya 2003 [54]	+	NR	?	?	NR	NR	+	+	+	+	NR	?	+	+	None
DeNysschen 2011 [55]	+	?	+	+	?	NR	+	+	+	+	+	+	+	?	None
Donnelly 2011 [24]	+	NR	NR	+	?	?	+	NR	?	?	NR	NR	?	NR	↓ Fatigue*
Edvardsen 2015 [56]	+	?	?	+	NR	NR	+	+	?	+	+	+	NR	NR	↑VO_2_peak*, leg press 1RM, stair climb, 30s sit-to-stand, BMI, total muscle mass, Tlco
Granger 2013 [57]	+	+	+	+	NR	NR	+	+	+	+	+	NR	NR	NR	↑, 6MWT
Hoffman 2017 [58]	+	+	NR	+	NR	NR	+	+	+	+	+	NR	NR	NR	↑ 6MWT
Kaibori 2013 [59]	+	NR	?	?	NR	NR	+	?	+	+	NR	NR	NR	NR	↑ VO_2_peak/AT VO_2_, platelet count test, branched-chain amino acid/tyrosine ratio (high frequency subgroup) ↓ BW, FM, insulin, insulin resistance
Onerup 2020 [60]	+	?	NR	+	?	?	+	+	+	+	+	?	?	+	None
Quist 2018 (EE) [61]	+	?	+	+	?	?	+	+	+	+	NR	NR	NR	NR	↑ VO_2_peak* (26 wks), ↑ 6MWT (14 wks) ↑ FEV1 (14, 26, 52 wks)
Quist 2018 (LE) [61]	+	?	+	+	?	?	+	+	+	+	NR	NR	NR	NR	↑ 6MWT ↑ FEV1 (26 wks)
Salhi 2015 [62]	+	NR	+	+	NR	NR	+	+	+	+	?	NR	NR	NR	↑ 6MWT*
Sommer 2016 (EE) [63]	+	+	+	+	?	?	+	+	+	+	?	?	?	?	None
Sommer 2016 (LE) [63]	+	?	+	?	?	?	+	+	+	+	+	?	NR	?	None
Stigt 2013 [64]	NR	NR	?	+	?	?	+	?	NR	?	+	NR	NR	NR	↑ 6MWT
*After treatment*															
Adams 2017 [65]	+	+	+	+	?	?	+	+	+	+	+	+	NR	?	↑ VO_2_peak *, HRR, respiratory sinus arrhythmia, carotid distensibility, brachial diameter, velocity time integral ↓ HR, DBP, carotid intima-media thickness, carotid-femoral PWV, femoral-toe PWV, CRP, LDL
Arbane 2014 [66]	+	?	+	+	NR	NR	+	+	?	+	NR	NR	NR	NR	↑ Leg strength (subgroup)
Bourke 2011 [67]	+	NR	NR	+	NR	NR	+	+	+	+	+	+	+	+	↑ Aer capacity, 30s sit-to-stand
Brocki 2014 [68]	+	+	+	+	?	?	+	+	+	+	NR	NR	NR	NR	None
Brown 2017 (high dose) [69]	+	+	+	+	NR	NR	?	+	+	+	+	+	+	+	↑ BMD, 6MWT ↓ Visceral adipose tissue, WC, sICAM-1
Brown 2017 (low dose) [69]	+	+	+	+	NR	NR	?	+	+	+	+	+	+	+	↑ BMD, 6MWT ↓ Insulin resistance, sICAM-1
Cavalheri 2017 [70]	+	?	+	+	NR	NR	+	+	+	+	NR	NR	NR	NR	↑VO_2_peak*, 6MWT
Chang 2020 [71]	+	NR	?	+	NR	NR	+	+	+	+	NR	NR	NR	NR	↑ VO_2_peak, 6MWT, albumin
Christensen 2019 [72]	+	?	+	+	?	?	?	+	+	+	?	+	+	+	↓ Glucose AUC, FM ↑ Matsuda index,
Crawford 2017 [73]	?	+	NR	+	NR	NR	+	NR	+	+	+	NR	NR	?	↑ 6MWT, 30s sit-to-stand, arm curl test, HGS, 8 ft. up-and-go, sit-and-reach
Devin 2016 (HIE) [74]	+	NR	+	NR	NR	NR	+	+	+	+	+	+	+	+	↑ VO_2_peak, PPO ↓ BW
Devin 2016 (MIE) [74]	+	NR	+	NR	NR	NR	+	+	+	+	+	+	+	+	None
Devin 2018 (HIIE) [75]	+	NR	+	?	+	?	+	+	+	+	+	+	+	+	↑ VO_2_peak ↓ FM
Devin 2018 (HIIE-T) [75]	+	NR	+	?	+	?	+	+	+	+	+	+	+	+	↑ VO_2_peak
Devin 2018 (MICE) [75]	+	NR	+	?	+	?	+	+	+	+	+	+	+	+	None
Gehring 2018 [76]	+	?	+	+	NR	NR	+	+	NR	+	+	+	+	+	None
Hausmann 2018 [77]	+	NR	NR	NR	?	?	+	?	+	?	NR	NR	NR	NR	↑ VO_2_peak
Lee 2013 [78]	+	?	NR	NR	NR	NR	?	?	?	+	?	?	?	NR	None
Lee 2017 [79]	?	?	NR	?	NR	NR	+	?	?	?	NR	NR	NR	NR	↓ Insulin*, TNF-α, ↑ 30s sit-to-stand, push-up, HGS
Lee 2018 [80]	?	NR	NR	?	NR	NR	+	NR	?	?	?	NR	NR	NR	↑ PA levels*, step test, push-up test
Lønbro 2013 (EE) [81]	+	?	NR	NR	?	NR	+	+	+	+	+	NR	NR	NR	↑ LBM, isometric knee ext., isokinetic knee flex (wk 12)
Lønbro 2013 (DE) [81]	+	?	NR	NR	?	NR	+	+	+	+	+	NR	NR	NR	↑ LBM, isometric knee ext./flex, isokinetic knee ext./flex, sit-to-stand, arm curl (wk 24)
Martin 2015 (HIG) [82]	+	?	+	NR	+	?	+	+	+	?	+	?	NR	NR	↑ VO_2_peak
Martin 2015 (LIG) [82]	+	?	+	NR	+	?	+	+	+	?	+	?	NR	NR	↑ VO_2_peak
Mascherini 2020 [83]	+	?	+	+	NR	?	+	?	+	?	?	NR	?	?	↑ 6MWT, 30s sit-to-stand, sit and reach ↓ BW, BMI, HC
McNeely 2008 [84]	+	+	+	+	NR	NR	+	+	+	+	+	NR	NR	NR	↓ Pain & disability* ↑ Chest press/seated row 1RM
Messaggi-Sartor 2019 [85]	+	+	+	+	NR	NR	+	+	+	+	+	NR	NR	+	↑ VO_2_ peak ↑ peak ventilation, max inspiratory pressure, max expiratory pressure, IGFBP-3
Meyerhardt 2020 [86]	+	+	?	+	NR	NR	?	?	+	+	?	?	?	?	↓ Insulin*, hs-CRP, IL6, insulin resistance, BW, BMI, WC
Nuri 2016 [87]	+	NR	NR	NR	+	?	+	?	+	?	NR	NR	NR	NR	↑ Ghrelin*, estimated VO_2_peak ↓ %BF
Pinto 2013 [88]	+	+	+	+	?	?	+	+	+	+	?	NR	+	NR	↑ PA levels*, estimated VO_2_peak*
Porserud 2014 [89]	+	NR	NR	NR	?	?	+	NR	+	?	+	NR	?	NR	↑ 6MWT*
Rossi 2016 [90]	+	?	?	+	NR	NR	+	+	+	+	+	NR	?	?	↑ 6MWT ↓ WC
Haematological cancer															
*During treatment*															
Alibhai 2015 [91]	+	+	+	+	?	?	+	?	+	+	+	NR	+	NR	↑ 6MWT, HGS, 10-chair stand test
Baumann 2010 [92]	+	NR	+	+	NR	NR	+	+	+	+	+	NR	NR	NR	↑ Aer capacity (W, min)*, knee ext.*, QoL*, IVC, FVC
Baumann 2011 [93]	+	NR	?	NR	NR	NR	+	+	+	+	+	NR	NR	NR	↑ Aer capacity (W/kg)*
Bryant 2018 [94]	+	+	?	+	NR	NR	+	+	+	+	+	NR	NR	NR	None
Coleman 2003 [95]	+	NR	?	NR	NR	NR	NR	NR	NR	+	+	NR	NR	NR	↑ LBM
Coleman 2012 [96]	+	NR	NR	+	NR	NR	?	+	+	+	?	NR	NR	NR	None
Duregon 2019 [97]	+	?	?	+	NR	NR	+	+	+	+	NR	NR	NR	NR	None
Jarden 2009 [98]	+	?	?	NR	?	NR	+	?	+	+	+	NR	NR	+	↑ Chest press/leg ext. 1RM, isometric knee ext
Larsen 2019 [99]	+	?	?	+	?	?	+	?	+	+	+	?	?	?	None
Oechsle 2014 [100]	+	NR	?	+	NR	NR	+	?	+	+	+	NR	NR	NR	↑ estimated VO_2_, VE
Santa-Mina 2020 [101]	+	?	+	+	?	?	+	?	+	+	?	?	?	?	None
Streckmann 2014 [102]	+	NR	?	+	?	?	+	+	+	+	?	NR	NR	?	↑ QoL*, peripheral deep sensitivity, balance control on static/dynamic surface & with perturbation
Wehrle 2019 (AER) [103]	+	NR	+	+	NR	NR	+	+	+	+	+	NR	NR	NR	None
Wehrle 2019 (RET) [103]	+	NR	NR	+	NR	NR	+	+	?	+	+	NR	NR	NR	↑ Knee ext./flex
*During/after treatment*															
Courneya 2009 [25]	+	+	+	NR	?	?	+	+	+	+	+	+	+	+	↑ QoL*, VO_2_peak, LBM ↓ %BF
Koutoukidis 2020 [104]	+	+	+	+	?	?	+	?	?	?	?	NR	?	?	↑ Leg ext
Mello 2003 [105]	?	?	NR	NR	NR	NR	+	+	+	+	NR	NR	NR	NR	↑ Hip flex*
Wiskemann 2011 [106]	+	?	+	+	?	?	+	+	+	+	+	NR	NR	NR	↑ 6MWT, lower body strength ↓ Total mortality (after discharge)
*After treatment*															
Alibhai 2014 [107]	+	NR	NR	+	?	?	+	?	+	+	+	NR	?	NR	None
Furzer 2016 [108]	+	+	+	NR	+	+	+	+	+	?	+	+	+	NR	↓ Fatigue* ↑ Aer capacity (W/kg), chest/arms/legs/total strength 1RM, %BF, LBM, BMD
Hacker 2011 [109]	+	?	?	+	NR	NR	+	+	+	+	+	NR	NR	NR	None
Hacker 2017 [110]	+	+	?	+	NR	NR	+	+	NR	+	+	NR	NR	NR	↑ Timed stair climb, TUG
Jarden 2013 [111]	+	?	?	+	NR	NR	+	+	+	+	+	NR	NR	NR	↑ 6MWT*, estimated VO_2_peak, 30s sit-to-stand, arm curl test
Knols 2011 [112]	+	?	?	?	?	?	+	+	+	+	+	NR	NR	NR	↑ 6MWT*, knee ext.*
Persoon 2017 [113]	+	+	+	+	NR	NR	+	+	+	+	+	NR	NR	NR	None
Shelton 2009 (Sup) [114]	+	?	NR	+	NR	NR	+	?	+	+	+	NR	NR	NR	None
Shelton 2009 (HB) [114]	+	?	NR	+	NR	NR	+	?	?	+	NR	NR	NR	NR	None
Mixed cancer types															
*During treatment*															
Adamsen 2009 [26]	+	?	+	NR	NR	NR	+	+	+	+	+	NR	NR	NR	↓ Fatigue* ↑ estimated VO_2_peak, leg press/chest press/pull down 1RM
Arrieta 2019 [115]	+	?	NR	+	?	?	?	?	?	?	?	?	?	?	↑ SPPB* (breast cancer, female, normal nutritional status subgroups only)
Griffith 2009 [116]	+	NR	?	+	NR	NR	+	+	+	+	+	NR	+	+	↑ VO_2_peak (prostate vs non-prostate)
Marechal 2019 [117]	+	?	?	+	NR	NR	+	+	+	+	NR	NR	NR	NR	↑ Sit-to-stand, global physical capacity score
Peterson 2018 [118]	+	+	?	+	NR	NR	+	+	+	+	NR	NR	NR	NR	None
Sturm 2014 [119]	?	NR	NR	+	NR	NR	+	NR	+	+	+	NR	NR	+	↑ 6MWT ↓ Fatigue*
Wenzel 2013 [120]	+	NR	+	+	NR	NR	+	+	+	+	NR	NR	NR	NR	↓ Sleep quality* ↑ Vigour
*During/after treatment*															
Courneya 2003 [121]	+	NR	?	NR	NR	NR	+	+	+	+	NR	?	+	NR	↑ QoL* ↓ %BF
Courneya 2008 [122]	+	NR	+	NR	NR	NR	+	+	NR	+	+	+	+	+	↑ VO_2_peak, PPO, VT
Irwin 2017 [123]	?	?	NR	NR	NR	NR	+	NR	?	?	+	NR	?	NR	↑ 6MWT
Mayo 2014 [124]	+	+	+	?	?	?	+	NR	+	+	+	NR	+	+	None
Schuler 2017 (Sup + HB) [125]	+	NR	NR	+	?	?	+	+	+	?	NR	NR	NR	NR	None
Schuler 2017 (HB) [125]	+	NR	NR	+	?	?	+	+	+	?	NR	NR	NR	NR	None
Schwartz 2009 (AER) [126]	+	NR	NR	+	NR	+	+	NR	+	+	+	NR	NR	NR	↓ Weight gain*, %BF* ↑ 12MWT, overhead press/seated row/leg press 1RM
Schwartz 2009 (RET) [126]	+	?	+	+	NR	+	+	NR	+	+	+	NR	NR	NR	None
*After treatment*															
Broderick 2013 [127]	+	+	+	+	+	+	+	+	+	+	+	+	+	NR	None
Burnham 2002 [128]	+	+	+	NR	NR	NR	+	+	+	+	+	NR	NR	NR	↑ VO_2_peak, flexibility ↓ %BF
Jones 2014 [129]	+	+	+	+	+	+	+	+	+	+	?	NR	+	+	↑ Cardiovascular mortality/hospitalization
Kampshoff 2015 (HI) [130]	+	+	+	?	NR	NR	+	+	+	+	+	?	+	+	↑ VO_2_peak *, PPO*, VT* (HI/LMI) ↓ Fatigue*
Kampshoff 2015 (LMI) [130]	+	+	+	?	NR	NR	+	+	+	+	+	?	+	+	None
Kneis 2019 (AER) [131]	+	?	+	+	NR	NR	+	+	+	+	+	NR	NR	NR	↑ Jump height/P_max_jump,_ vibration sense
Kneis 2019 (AER + balance) [131]	+	?	+	+	NR	NR	+	+	+	+	+	NR	NR	NR	↑ MS_EOunstable_ duration, patella vibration ↓ ST_EO_
Knobf 2017 [132]	+	?	+	+	NR	NR	+	+	+	+	+	NR	?	NR	↑ Aer capacity, HRR ↔ Insulin
LaStayo 2011 [133]	+	+	NR	+	NR	NR	+	+	+	+	+	+	NR	+	↑ Muscle CSA, 6MWT, stair descent
Midtgaard 2013 [134]	+	+	+	+	NR	NR	+	+	+	+	+	?	NR	NR	↑ PA levels*, VO_2_peak*, leg press/chest press 1RM
Pisu 2017 [135]	+	NR	NR	+	NR	NR	+	+	+	+	+	NR	NR	NR	None
Thorsen 2005 [136]	?	NR	?	NR	NR	NR	+	+	+	+	+	NR	NR	+	↑ VO_2_peak*
Toohey 2016 (LVHIIT) [137]	+	+	+	+	NR	NR	+	+	+	+	?	?	?	+	↑ 6MWT
Toohey 2016 (CLMIT) [137]	+	NR	+	+	NR	NR	+	+	+	+	?	?	?	+	None

*Primary outcome where specifically stated, +: clear reporting, *NR* not reported,?: unclear reporting, *%BF* body fat %, *1RM* 1-repetition maximum, *12MWT* 12-min walk test, *6MWT* 6-min walk test, *AER* aerobic exercise, *BM* body mass, *BMD* bone mineral density, *BP* blood pressure, *BW* body weight, *CG* control group, *CRP* C-reactive protein, *CSA* cross-sectional area, *DE* delayed exercise, *EE* early exercise, *FM* fat mass, *FVC* forced vital capacity, *HC* hip circumference, *hs-CRP* high-sensitivity C-reactive protein, *HGS* handgrip strength, *HI* high intensity exercise, *HIE* high-intensity exercise, *HIG* high-intensity group, *HIIE-T* high-intensity interval exercise-tapered, *HR* heart rate, *HRR* heart rate recovery, *IG* intervention group, *IL6* interleukin 6, *IVC* inspiratory vital capacity, *LBM* lean body mass, *LDL* low-density lipoprotein, *LIG* low-intensity group, *LMI* low-to-moderate intensity exercise, *LVHIIT* low-volume high intensity interval training, *MAP* mean arterial pressure, *MICE* moderate intensity continuous exercise, *MIPT* maximum isokinetic peak torque, *MSEO* monopedal stance on stable surface, *MSEOunstable* monopedal stance on unstable surface, *MVIC* maximum voluntary isometric contraction, *Pmax_jump* maximum jump power output, *PPO* peak power output, *PWV* pulse wave velocity, *QoL* quality of life, *RET* resistance training, *ROM* range of motion, *RPP* rate pressure product, *SPPB* short physical performance battery, *STEO* semi-tandem stance with eyes open, *Sup* supervised, *TC* total cholesterol, *TG* triglycerides, *Tlco* carbon monoxide transfer factor, *TUG* timed up and go, *VE* ventilatory equivalent, *VO*_*2*_ oxygen consumption, *VO*_*2*_*peak* peak oxygen consumption, *VT* ventilatory threshold, *W* watts, *WC* waist circumference

These corrections were typesetting errors and do not influence the results of this article. The publishers apologise for this error. The original article [1] has been updated.
