# Supplementary material for: Attention to principles of exercise training: an updated systematic review of randomized controlled trials in cancers other than breast and prostate
Source: BMC Cancer. 2021 Nov 5;21:1179. doi: 10.1186/s12885-021-08701-y (PMC8569988; doi:10.1186/s12885-021-08701-y)
Supplement: Supplementary file 1 — Additional file 1: Table S1. Description of studies. [file 12885_2021_8701_MOESM1_ESM.docx]

| **Table: Description of studies** | | | | | | | | | | | | | | | | | | | | | | | | | | | | | | | |  |
| --- | --- | --- | --- | --- | --- | --- | --- | --- | --- | --- | --- | --- | --- | --- | --- | --- | --- | --- | --- | --- | --- | --- | --- | --- | --- | --- | --- | --- | --- | --- | --- | --- |
| **Studies in solid tumours** | | | | | | | | | | | | | | | | | | | | | | | | | | | | | | | |  |
| **Reference** | | **Cancer** | | | | **Treatment** | | | | | **N** | | | **Follow-Up** | | | **Intervention** | | | | **Length** | | | | **Primary Outcome*** | | | **Secondary Fitness & Physiological Outcomes** | | | |  |
| ***During treatment*** | | | |  | | | |  | | | | | | |  | | |  | | | | |  | | |  | | | |  |  |  |
| Arbane 2011 | | Lung | | | | S/CT | | | | | 53 | | | Post-only | | | Sup + HB AER RET | | | | 12 wks | | | | 6MWT, strength (magnetic stimulation) | | | BMI, length of stay, post-op complications | | | |  |
|  | |  | | | |  | | | | |  | | |  | | |  | | | |  | | | |  | | |  | | | |  |
| Backman 2014 | | Breast & Colorectal | | | | CT | | | | | 71 | | | Post-only | | | Sup + HB AER | | | | 10 wks | | | | Feasibility | | | BW, BMI, WC, BP, HR, HDL, LDL, TC, C-peptide | | | |  |
|  | |  | | | |  | | | | |  | | |  | | |  | | | |  | | | |  | | |  | | | |  |
| Capozzi 2016 | | Head & Neck | | | | RT/CRT | | | | | 60 | | | 12, 24 wks | | | Sup + HB RET | | | | 12 wks | | | | BMI, LBM, %BF (DXA) | | | 6MWT, strength (30s sit-to-stand, HGS), flexibility (sit-and-reach) | | | |  |
|  | |  | | | |  | | | | |  | | |  | | |  | | | |  | | | |  | | |  | | | |  |
| Christensen 2014 | | Testicular | | | | CT | | | | | 30 | | | 9, 21 wks | | | Sup RET | | | | 9 wks | | | | Muscle fibre size (CSA) | | | LBM, %BF (DXA), strength (isometric force, 1-RM), HDL, LDL, TC, glucose, insulin, plasma granulocyte macrophage colony-stimulating factor, interferon-ƴ, IL-1β, IL-2, IL-6, IL-8, IL-10, IL-12, TNF-α, muscle phenotype distribution, muscle capillary density, satellite cell activation, mRNA expression | | | |  |
| Grote 2018 | | Head & Neck | | | | RT/CRT | | | | | 20 | | | 7-8 wks | | | Sup RET | | | | ~6 wks | | | | Feasibility | | | BW, LBM, FM (BIA), 6MWT, strength (isometric force) | | | |  |
|  | |  | | | |  | | | | |  | | |  | | |  | | | |  | | | |  | | |  | | | |  |
| Hammer 2020 | | Breast, Lung, Gastrointestinal, & Gynaecological | | | | CT | | | | | 33 | | | Post-only | | | HB AER | | | | 24 wks | | | | Feasibility | | | 6MWT, glycaemic control (HbA1c) | | | |  |
| Kamel 2020 | | Pancreatic | | | | CT | | | | | 40 | | | Post-only | | | Sup RET | | | | 12 wks | | | | NR | | | LBM, %BF (DXA), 6MWT, strength (isokinetic torque, isometric force, 30s sit-to-stand), 400m walk | | | |  |
| Lin 2014 | | Colorectal | | | | CT | | | | | 45 | | | Post-only | | | Sup AER + RET | | | | 12 wks | | | | QoL (QLQ-C30) | | | 6MWT, strength (isometric force, HGS) | | | |  |
| Moller 2015 | | Breast & Colon | | | | CT | | | | | 45 | | | 39 wks | | | Sup + HB AER + RET | | | | 12 wks | | | | Aer capacity (VO_2_peak) | | | None | | | |  |
| Mustian 2009 | | Breast & Prostate | | | | RT | | | | | 38 | | | 4, 16 wks | | | HB AER + RET | | | | 4 wks | | | | Fatigue (BFI, FACIT-F) | | | LBM (BIA), 6MWT, strength (HGS), IL-6, TNF-α, sTNF-R | | | |  |
| Rogers 2013 | | Head & Neck | | | | RT | | | | | 15 | | | 6, 12 wks | | | Sup + HB RET | | | | 12 wks | | | | Feasibility | | | BMI, LBM (BIA), strength (isometric force, HGS), SPPB | | | |  |
| Samuel 2013 | | Head & Neck | | | | CRT | | | | | 48 | | | Post-only | | | HB AER + RET | | | | 6 wks | | | | 6MWT, QoL (SF-36) | | | NR | | | |  |
| Samuel 2019 | | Head & Neck | | | | CRT | | | | | 148 | | | 3, 7, 11 wks | | | Sup + HB AER + RET | | | | 11 wks | | | | 6MWT, QoL (SF-36) | | | Hb, Plts | | | |  |
| Sandmael 2017 | | Head & Neck | | | | CRT/RT | | | | | 41 | | | 6, 14 wks | | | Sup RET | | | | 6 wks | | | | Feasibility | | | BW, LBM, regional adipose tissue, radiodenisty of skeletal mass (computed tomography) | | | |  |
| Stuecher 2019 | | Gastrointestinal | | | | CT | | | | | 44 | | | ~6, 12 wks | | | HB AER | | | | 12 wks | | | | SPPB | | | LBM (BIA), gait speed (five stride time), postural stability (COP) strength (isometric force), peripheral deep sensitivity (tuning fork) | | | |  |
| VanVulpen 2016 | | Colon | | | | CT | | | | | 22 | | | 18, 36 wks | | | Sup + HB AER + RET | | | | 18 wks | | | | Fatigue (MFI, FQL) | | | BW, BMI, Aer capacity (VO_2_peak, PPO, VT), chemotherapy completion rates | | | |  |
| Vigario 2011 | | Thyroid | | | | TSH-S | | | | | 36 | | | Post-only | | | Sup AER | | | | 12 wks | | | | NR | | | BW, BMI, girth measurements, WHR, %BF, sum, LBM, bone mass, residual mass, muscle mass (SkF) | | | |  |
| Xu 2015 | | Oesophagus | | | | CRT | | | | | 56 | | | Post-only | | | Sup AER | | | | 4-5 wks | | | | BW, LBM (BIA), 6MWT | | | Strength (HGS), chemotherapy completion rate | | | |  |
| Yen 2019 | | Head & Neck | | | | CT | | | | | 84 | | | Post-only | | | Sup + HB AER + RET | | | | 8 wks | | | | 6MWT | | | BW, BMI, %BF, visceral fat, skeletal muscle rate (method NR), BP, MAP, HR, SPO_2_, RPP, RPE (rest, during & post 6MWT) | | | |  |
| Zhao 2016 | | Head & Neck | | | | CRT | | | | | 20 | | | 7, 14 wks | | | Sup + HB AER + RET | | | | 14 wks | | | | NR | | | BMI, %LBM (DXA), 6MWT, TUG, strength (isokinetic force, HGS) | | | |  |
| ***During/after treatment*** | | | | | | | | | | | | | | | | | | | | | | | | | | | | | | | |  |
| Courneya 2003 | | Colorectal | | | | S/CT/RT | | | | | 102 | | | Post-only | | | HB AER | | | | 16 wks | | | | QoL (FACT-C) | | | BMI, sum of SkF, Aer capacity (GXT, time), flexibility (sit-and-reach), HR | | | |  |
| DeNysschen 2011 | | Breast, colorectal, ovarian | | | | CT/RT | | | | | 119 | | | Post-only | | | HB AER | | | | 12 mo | | | | Fatigue (PFS) | | | BW, LBM, FM, %BF (DXA), Aer capacity (VO_2_peak) | | | |  |
| Donnelly 2011 | | Gynaecological | | | | S/CT | | | | | 33 | | | 12, 24 wks | | | HB AER + RET + behavioural | | | | 12 wks | | | | Fatigue (MFSI, FACIT-F) | | | BMI, WC, 12MWT | | | |  |
| Edvardsen 2015 | | Lung | | | | S/CT/RT | | | | | 61 | | | Post-only | | | Sup AER + RET | | | | 20 wks | | | | Aer capacity (CPET) | | | Total muscle mass (DXA), strength (1-RM, HGS), 15s stair reps, 30s sit-to-stand, balance, dyspnoea, spirometry | | | |  |
| Granger 2013 | | Lung | | | | S/CT | | | | | 15 | | | Post-only | | | Sup AER + RET | | | | 12 wks | | | | Safety, feasibility | | | 6MWT, TUG | | | |  |
| Hoffman 2017 | | Lung | | | | S/CT | | | | | 73 | | | Post-only | | | HB AER | | | | 6 wks | | | | Feasibility | | | 6MWT | | | |  |
| Kaibori 2013 | | Liver | | | | S | | | | | 51 | | | Post-only | | | HB AER | | | | 4 wks pre-op, 24 wks post-op | | | | NR | | | BMI, FM, non-mineral FFM, truncal fat, BMD (DXA), Aer capacity (VO_2_peak, AT VO_2_), liver function, albumin, Plt, ALT, insulin, glucose, HOMA-IR, BCAA, AAA, BTR, tumour size, number of tumours, histology, microscopic capsule formation, microscopic vascular invasion, microscopic survival margin <5mm from tumour border, liver diseases, tumour-node-metastasis stage, surgical procedure, operating time, operative blood loss, blood transfusion, length of hospitalization | | | |  |
| Onerup 2020 | | Colorectal | | | | S/RT | | | | | 112 | | | Up to 2 wks post-intervention | | | HB AER | | | | 6 wks (2 wks pre-op, 4 wks post-op) | | | | Self-assessed physical recovery | | | IGF-1, IGFBP-3, HbA1c | | | |  |
| Quist 2018 | | Lung | | | | S/CT | | | | | 235 | | | 14, 26, 52 wks | | | Sup AER + RET | | | | 12 wks | | | | Aer capacity (VO2peak) | | | 6MWT, strength (1-RM), lung function (FEV1) | | | |  |
| Salhi 2015 | | Lung | | | | S/CT/RT | | | | | 70 | | | Post-only | | | Sup AER + RET | | | | 12 wks | | | | 6MWT | | | Aer capacity (VO_2_max, Wmax), strength (HGS, leg), fatigue, pain, dyspnoea, spirometry, diffusion capacity | | | |  |
| Sommer 2016 | | Lung | | | | S/CT | | | | | 57 | | | 18, 26, 52 wks | | | Sup HB AER + RET | | | | 12 wks | | | | Safety, feasibility | | | Aer capacity (VO_2_peak, O_2_ delivery), 6MWT, strength (1-RM) lung function (FEV1, FVC) | | | |  |
| Stigt 2013 | | Lung | | | | S/CT | | | | | 49 | | | 12, 24, 52 wks | | | Sup AER + RET | | | | 12 wks | | | | QoL (SGRQ) | | | 6MWT, lung function (FEV1, FVC) | | | |  |
| ***After treatment*** | | | | | | | | | | | | | | | | | | | | | | | | | | | | | | | |  |
| Adams 2017 | | Testicular | | | | Mixed | | | | | 63 | | | Post-only | | | Sup AER | | | | 12 wks | | | | Aer capacity (VO_2_peak) | | | HR, BP, HRR, respiratory sinus arrhythmia, carotid intima-media thickness/distensibility/diameter (ultrasound), brachial diameter/FMD (%, normalized), velocity time integral, shear stress during reactive hyperaemia, carotid-femoral PWV, femoral-toe PWV, fibrinogen, CRP, HDL, LDL, TC, TC:HDL ratio, TG, glucose, testosterone | | | |  |
| Arbane 2014 | | Lung | | | | S | | | | | 131 | | | Post-only | | | Sup + HB AER RET | | | | 4 wks | | | | Physical activity (actigraphy) | | | Strength (isokinetic force), shuttle walk, length of stay, post-op complications | | | |  |
| Bourke 2011 | | Colon | | | | S/CT | | | | | 18 | | | Post-only | | | Sup + HB AER + RET | | | | 12 wks | | | | Feasibility | | | Aer capacity (GXT, RPE), strength (isokinetic torque, 30s sit-to-stand), muscle fatigue (EMG), | | | |  |
| Brocki 2014 | | Lung | | | | S | | | | | 78 | | | 16, 52 wks | | | Sup +HB AER RET | | | | 10 wks | | | | QoL (SF-36) | | | 6MWT, lung function (FEV1, FVC, FEV1/FVC) | | | |  |
| Brown 2017 | | Colon | | | | S/CT | | | | | 39 | | | Post-only | | | HB AER (high dose) HB AER (low dose) | | | | 24 wks | | | | Feasibility | | | BW, BMI, WC, HC, WHR, sagittal abdominal diameter, LBM, FM, visceral/subcutaneous adipose tissue, BMD (DXA), 6MWT, glucose, insulin, HbA1c, IGF-1, IGFBP3, C-peptide, fructosamine, sICAM-1, sVCAM-1, circulating tumour cells | | | |  |
| Cavalheri 2017 | | Lung | | | | S/CT | | | | | 17 | | | Post-only | | | Sup AER RET | | | | 8 wks | | | | Aer capacity (VO_2_peak, VO_2_ at AT, Wmax) | | | 6MWT, strength (HGS) | | | |  |
| Chang 2020 | | Oesophageal | | | | S | | | | | 88 | | | Post-only | | | HB AER | | | | 12 wks | | | | QoL (QLQ-C30, QLQ OES-18) | | | Aer capacity (VO_2_peak), 6MWT, albumin, BMI | | | |  |
| Christensen 2019 | | Colorectal | | | | S/CT | | | | | 39 | | | Post-only | | | HB AER | | | | 12 wks | | | | Aer capacity (VO_2_peak) | | | BC (DXA), glycaemic control (oral glucose tolerance test), blood biochemistry (lipid concentrations, plasma leptin, cytokine levels) | | | |  |
| Crawford 2017 | | Gynaecological | | | | S/CT/RT | | | | | 35 | | | Post-only | | | Sup Wall Climbing | | | | 8 wks | | | | Feasibility | | | BW, BMI, WC, 6MWT, 8ft up-and-go, strength (30s sit-to-stand, arm curl test, HGS), flexibility (sit-and-reach) | | | |  |
| Devin 2016 | | Colorectal | | | | S/CT/RT | | | | | 47 | | | Post-only | | | Sup MIE Sup HIIE | | | | 4 wks | | | | NR | | | BW, LBM, FM, %BF (DXA), Aer capacity (VO₂peak, PPO) | | | |  |
| Devin 2018 | | Colorectal | | | | S/CT/RT | | | | | 57 | | | 4, 8, 12 wks | | | Sup MICE Sup HIIE Sup HIIE-T | | | | 8 wks | | | | Aer capacity (VO_2_peak) | | | LBM, FM (DXA) | | | |  |
| Gehring 2018 | | Brain (gliomas) | | | | S/CT/RT | | | | | 34 | | | Post-only | | | HB AER | | | | 24 wks | | | | Feasibility | | | BW, BMI, Aer capacity (VO_2_peak), cognitive function (neuropsychological testing) | | | |  |
| Hausmann 2018 | | Gynaecological | | | | S/CT/RT | | | | | 60 | | | 52 wks | | | Sup AER + RET | | | | 16 wks | | | | NR | | | Aer capacity (VO_2_peak), strength (1-RM) | | | |  |
| Lee 2013 | | Colorectal | | | | S/CT | | | | | 23 | | | Post-only | | | HB AER | | | | 12 wks | | | | PA levels (IPAQ) | | | BW, WC, %BF, LBM, FM (BIA), insulin, glucose, HbA1c, HOMA-IR, TC, TG, HDL, LDL, hs-CRP, WBC, Hb, TNF-α, IL-6, leptin, osteopontin, adiponectin, IGFBP-1, IGF-1, IGFBP-3 | | | |  |
| Lee 2017 | | Colorectal | | | | S/CT/RT | | | | | 123 | | | Post-only | | | HB AER + RET | | | | 12 wks | | | | Insulin | | | BW, WC, 6MWT, strength (30s sit-to-stand, push-up test, HGS), glucose, HOMA-IR, TC, TG, HDL, hs-CRP, adiponectin, TNF-α | | | |  |
| Lee 2018 | | Colorectal | | | | S/CT/RT | | | | | 72 | | | Post-only | | | HB AER + RET | | | | 6 wks | | | | PA levels (Godin) | | | BMI, WC, %BF (BIA), 6MWT, Tecumseh step test, strength (30s sit-to-stand, push-up test) | | | |  |
| Lonbro 2013 | | Head & Neck | | | | RT | | | | | 41 | | | 12, 24 wk | | | Unsup RET (EE) Unsup RET (DE) | | | | 12 wks | | | | LBM (DXA) | | | 10m gait speed, strength (isokinetic torque, isometric force, 30s sit-to-stand, arm curl test), max stair climb | | | |  |
| Martin 2015 | | Breast & Prostate | | | | S/CT/RT | | | | | 159 | | | 8, 24 wks | | | Sup AER + RET (HIG) Sup AER + RET (LIG) | | | | 8 wks | | | | Aer capacity (VO_2_peak) | | | None | | | |  |
| Mascherini 2020 | | Colorectal | | | | S | | | | | 6 | | | Post-discharge; 30d, 90-100d, 180d post-discharge | | | Sup + HB AER + RET | | | | 24 wks | | | | NR | | | BC (BIA), 6MWT, strength (30s sit-to-stand, HGS), flexibility (sit-and-reach) | | | |  |
|  | |  | | | |  | | | | |  | | |  | | |  | | | |  | | | |  | | |  | | | |  |
| McNeely 2008 | | Head & Neck | | | | S | | | | | 52 | | | Post-only | | | Sup RET | | | | 12 wks | | | | Shoulder function (SPADI) | | | Strength (1-RM), muscle endurance (reps*kg), flexibility (ROM) | | | |  |
| Messaggi-Sartor 2019 | | Lung | | | | S | | | | | 37 | | | 24 wks | | | Sup AER + RET | | | | 8 wks | | | | Aer capacity (VO_2_peak) | | | Respiratory muscle strength, serum IGF-I and IGFBP-3, cancer recurrence, death | | | |  |
| Meyerhardt 2020 | | Breast & Colorectal | | | | S/CT/RT | | | | | 139 | | | Post-only | | | Sup + HB AER  Sup + HB AER + metformin | | | | 12 wks | | | | Fasting plasma insulin | | | BM, HC/WC, 6MWT, insulin resistance, leptin, IGF-1, glucose, hs-CRP, sTNFaR2, IL6 | | | |  |
| Nuri 2016 | | Colorectal | | | | S | | | | | 30 | | | Post-only | | |  | | | | 8 wks | | | | Leptin, ghrelin | | | BW, BMI, %BF (BIA), Aer capacity (estimated VO2peak), glucose, insulin, HOMA-IR | | | |  |
| Pinto 2013 | | Colorectal | | | | Mixed | | | | | 46 | | | Post-intervention, 24, 52 wks | | | HB AER | | | | 12 wks | | | | PA levels (7-day PAR), Aer capacity (estimated VO_2_peak) | | | BMI, FFM, FM (BIA) | | | |  |
| Porserud 2014 | | Bladder | | | | S | | | | | 18 | | | 12, 52 wks | | | Sup + HB AER + RET | | | | 12 wks | | | | 6MWT | | | Strength (30s sit-to-stand), balance (figure eight walking) | | | |  |
| Rossi 2016 | | Endometrial | | | | Mixed | | | | | 40 | | | Post-only | | | Sup + HB AER + RET | | | | 12 wks | | | | Feasibility | | | BW, WC, BMI, 6MWT, strength (30s sit-to-stand) | | | |  |
| **Studies in haematological cancers** | | | | | | | | | | | | | | | | | | | | | | | | | | | | | | | |  |
| **Reference** | **Cancer** | | | | **Treatment** | | | | | **N** | | **Follow-Up** | | | | **Intervention** | | | | **Length** | | | | **Primary Outcome*** | | | | | **Secondary Fitness & Physiological Outcomes** | | |  |
| ***During treatment*** | | | | | | | | | | | | | | | | | | | | | | | | | | | | | | | |  |
| Alibhai 2015 | AML | | | | CT | | | | 81 | | | ~4, 8-12 wks, | | | | Sup AER + RET | | | | ~4 wks | | | | NR | | | | | BMI, Aer capacity (VO_2_peak), 6MWT, strength (10-chair stand test, HGS), HR, BP, SpO_2_, length of admission, occurrence of sepsis, ICU admission, time to start 2^nd^ chemotherapy cycle | | |  |
| Baumann 2010 | Mixed | | | | SCT | | | | 64 | | | Post-only | | | | Sup AER | | | | ~6 wks | | | | Aer capacity (submax GXT, W, W/kg, min), strength (isometric force), QoL (QLQ-C30) | | | | | Lung function (IVC, FVC), leucocytes, Plts, Hb, engraftment (days) | | |  |
| Baumann 2011 | Leukemia | | | | CT/SCT | | | | 45 | | | Post-only | | | | Sup AER | | | | Pre-SCT, during CT, post-engraftment | | | | Aer capacity (GXT, W, min, W/kg) | | | | | BW, BMI, strength (isometric force), lung function (FVC, VC) | | |  |
| Bryant 2018 | Leukemia | | | | CT | | | | 18 | | | Post-only | | | | Sup AER + RET | | | | 4-6 wks | | | | Fatigue (PROMIS), QoL (SF-12v2), TUG, 6MWT | | | | | Strength (HGS, composite strength) | | |  |
| Coleman 2003 | Multiple myeloma | | | | CT/SCT | | | | 24 | | | ~10, 24 wks | | | | HB AER + RET | | | | ~24 wks | | | | NA | | | | | LBM (Bodpod), Aer capacity (GXT, time), strength (1-RM) | | |  |
| Coleman 2012 | Multiple myeloma | | | | CT/SCT | | | | 135 | | | Pre-CT, pre-SCT, post-SCT | | | | HB AER + RET | | | | 30 wks | | | | Response to EPO | | | | | 6MWT, total RBC/platelet transfusions, Hb | | |  |
| Duregon 2019 | AML, lymphoma, multiple myeloma | | | | CT | | | | 42 | | | Post-only | | | | Sup RET | | | | 3-4 wks | | | | Feasibility | | | | | Strength (30s sit-to-stand, arm curl test, HGS) flexibility (back scratch, sit-and-reach), balance (FAB) | | |  |
| Jarden 2009 | Mixed Haematological | | | | SCT | | | | 42 | | | Post-only | | | | Sup AER + RET | | | | 4-6 wks | | | | Symptoms (24-item questionnaire) | | | | | Aer capacity (estimated VO_2_peak), strength (estimated 1-RM, isometric force), 2-min stair climb | | |  |
| Larsen 2019 | Multiple myeloma | | | | SCT | | | | 24 | | | 11, 24, 52 wks | | | | Sup + HB AER + RET | | | | 10 wks | | | | Static knee extensor strength (dynamometer) | | | | | 6MWT, strength (HGS, 30s sit-to-stand), bone disease (DXA) | | |  |
| Oechsle 2014 | AML | | | | CT | | | | 48 | | | Post-only | | | | Sup AER + RET | | | | ~21 days | | | | Aer capacity (GXT, Lac, VO_2_, VCO_2_, VE, RQ) | | | | | BW, BMI, pulmonary function (spirometry), muscular strength (1-RM), days of hospital stay, number of RBC or Plt transfusions | | |  |
| Santa-Mina 2020 | Leukemia, lymphoma, myelodysplastic syndrome | | | | CT/SCT | | | | 30 | | | Median 13.5 wks | | | | Sup + HB AER + RET | | | | Median 5 wks (prehab), 4 wks (inpatient), 13.5 wks (post-discharge) | | | | Feasibility | | | | | BMI, %BF (BIA), Aer capacity (VO_2_peak), 6MWT, strength (HGS, 30s sit-to-stand, isometric force) | | |  |
| Streckmann 2014 | Lymphoma | | | | CT | | | | 61 | | | 12, 24, 36 wks | | | | Sup AER + RET (+ balance) | | | | 36 wks | | | | QoL (QLQ-C30) | | | | | Aer capacity (GXT, LT, IAT), balance control (force plate), peripheral deep sensitivity (tuning fork) | | |  |
| Wehrle 2019 | AL | | | | CT | | | | 29 | | | Post-only | | | | Sup AER  Sup RET | | | | ~5 wks | | | | NR | | | | | Aer capacity (IAT, PPO), strength (isokinetic torque), standardized phase angle (BIA), Hb, thrombocytes, leucocytes, hospitalization (days) | | |  |
| ***During/after treatment*** | | | | | | | | | | | | | | | | | | | | | | | | | | | | | | | |  |
| Courneya 2009 | Lymphoma | | | | CT/RT | | | | 122 | | | 24 wks | | | | Sup AER | | | | 12 wks | | | | QoL (FACT-An) | | | | | LBM, FM, %BF (DXA), Aer capacity (VO_2_peak), | | |  |
| Koutoukidis 2020 | Multiple myeloma | | | | S/RT/SCT | | | | 83 | | | 12, 24, 52 wks | | | | Sup + HB AER + RET | | | | 24 wks | | | | Fatigue (FACIT-F) | | | | | BW, %BF (BIA), Aer capacity (VO_2_peak), strength (leg extension test, HGS) haematology/biochemistry blood tests | | |  |
| Mello 2003 | Mixed Haematological | | | | BMT | | | | 18 | | | Pre & post-BMT, 6 wks | | | | Sup AER | | | | 6 wks | | | | Strength (isometric force) | | | | | None | | |  |
| Wiskemann 2011 | Mixed Haematological | | | | SCT | | | | 105 | | | Discharge, 6-8 wks post-discharge | | | | Sup + HB AER + RET | | | | 7-12 wks | | | | Fatigue (MFI, POMS) | | | | | 6MWT, strength (isometric force, HGS), non-relapse mortality, total mortality | | |  |
| ***After treatment*** | | | | | | | | | | | | | | | | | | | | | | | | | | | | | | | |  |
| Alibhai 2014 | AML | | | | SCT | | | | 40 | | | 12, 18, 24 wks | | | | Sup + HB AER + RET | | | | 12 wks | | | | Feasibility | | | | | BMI, %BF (SkF), 6MWT, strength (HGS), flexibility (sit-and-reach), HR, BP | | |  |
| Furzer 2016 | Mixed Haematological | | | | CT | | | | 44 | | | 12, 24 wks | | | | HB AER + RET | | | | 12 wks | | | | Fatigue (SCFS, SF-36 vitality subscale, Aer power index) | | | | | %BF, LBM, BMD (DXA), Aer capacity (submax GXT, W/kg, 75% VO_2_/kg), strength (1-RM) | | |  |
| Hacker 2011 | Mixed Haematological | | | | SCT | | | | 19 | | | During SCT, 6 wks post-discharge | | | | Sup + HB RET | | | | 6 wks | | | | NR | | | | | Strength (30s sit-to-stand, timed stair climb, HGS), timed up from bed rest exam | | |  |
| Hacker 2017 | Mixed Haematological | | | | SCT | | | | 67 | | | Post-only | | | | Sup + HB RET | | | | 6 wks | | | | NR | | | | | 15ft walk test, TUG, strength (30s sit-to-stand, arm curl test, timed stair climb, HGS), muscle CSA (ultrasound) | | |  |
| Jarden 2013 | Leukemia | | | | CT | | | | 70 | | | 6, 12 wks | | | | Sup AER + RET | | | | 12 wks | | | | 6MWT | | | | | BW, BMI, Aer capacity (estimated VO_2_peak), strength (30s sit-to-stand, arm curl test), elevated CRP (days), hospitalization (days), neutropenia/thrombocytopenia (days), RBC/Plts transfusions (number) | | |  |
| Knols 2011 | HSCT | | | | SCT | | | | 131 | | | 12, 24 wks | | | | Sup AER + RET | | | | 12 wks | | | | Physical performance (isometric force, HGS, 6MWT, QLQ-C30 PF subscale) | | | | | BW, BMI, FFM, FM (DXA) | | |  |
| Persoon 2017 | Multiple myeloma or lymphoma | | | | CT/RT/SCT | | | | 109 | | | Post-only | | | | Sup AER + RET | | | | 18 wks | | | | Aer capacity (VO_2_peak, PPO), strength (30s sit-to-stand, HGS), fatigue (MFI) | | | | | BMI, sum of Skf, strength (isometric force) | | |  |
| Shelton 2009 | Lymphoma & leukemia | | | | SCT | | | | 53 | | | Post-only | | | | Sup AER + RET HB AER + RET | | | | 4 wks | | | | NR | | | | | 6MWT, 50ft walk test, strength (2x sit-to-stand), balance (uniped stance time), flexibility (forward reach) | | |  |
| **Studies in mixed cancer types** | | | | | | | | | | | | | | | | | | | | | | | | | | | | | | | | |
| **Reference** | | | **Treatment** | | **N** | | **Follow-Up** | | | | | | **Intervention** | | | | | | **Length** | | | **Primary Outcome*** | | | | | **Secondary Fitness & Physiological Outcomes** | | | | | |
| ***During treatment*** | | | | | | | | | | | | | | | | | | | | | | | | | | | | | | | | |
| Adamsen 2009 | | | CT | | 269 | | Post-only | | | | | | Sup AER + RET | | | | | | 6 wks | | | Fatigue (QLQ-C30) | | | | | Aer capacity (estimated VO_2_peak), strength (1-RM) | | | | | |
| Arrieta 2019 | | | Mixed | | 301 | | 24, 52, 104 wks | | | | | | HB AER + RET | | | | | | 52 wks | | | SPPB (decline ≥1) | | | | | BMI, strength (HGS), CRP | | | | | |
| Griffith 2009 | | | CT/RT | | 126 | | Post-only | | | | | | HB AER | | | | | | ~10-16 wks | | | Aer capacity (estimated VO₂peak) | | | | | None | | | | | |
| Marechal 2019 | | | Mixed | | 14 | | None | | | | | | Sup + HB AER + RET | | | | | | 12 wks | | | NR | | | | | BW, WC, BMI, 8ft up-and- go, 6MWT, strength (arm curl test, 30s sit-to-stand, HGS, 1-RM), flexibility (sit-and-reach), global physical capacity score (from PF sit-to-stand) | | | | | |
| Peterson 2018 | | | Mixed | | 28 | | Post-only | | | | | | Sup AER  Sup AER + Cog Training | | | | | | 12 wks | | | Cognitive function (neuropsychological testing) | | | | | BW, BMI, BC (Skf), circumference measures, Aer capacity (estimated VO_2_peak), strength (estimated 1-RM, HGS), muscular endurance (cable assisted machines, chair squat test, plank test), flexibility (sit-and-reach, shoulder reach behind back), balance, pulmonary function (spirometry), HR, BP, SpO_2_ | | | | | |
| Sturm 2014 | | | CT/RT/HT | | 40 | | Post-only | | | | | | Sup AER (Dance) | | | | | | 5 wks | | | Fatigue (FACIT-F, NRS) | | | | | 6MWT | | | | | |
| Wenzel 2013 | | | CT/RT | | 138 | | Post-only | | | | | | HB AER | | | | | | 5-35 wks | | | Sleep quality (PSQI), distress (POMS), fatigue (PFS), PA levels (PAQ) | | | | | Aer capacity (VO_2_peak) | | | | | |
| ***During/after treatment*** | | | | | | | | | | | | | | | | | | | | | | | | | | | | | | | | |
| Courneya 2003 | | | S/CT/RT | | 108 | | Post-only | | | | | | HB AER + psychotherapy | | | | | | 10 wks | | | QoL (FACT-G) | | | | | BW, sum of SkF, Aer capacity (GXT, time), flexibility (sit-and-reach) | | | | | |
| Courneya 2008 | | | CT/RT | | 55 | | Post-only | | | | | | Sup AER | | | | | | 12 wks | | | NR | | | | | Aer capacity (VO_2_peak, PPO, VT), Hb, darbepoetin alfa dosage | | | | | |
| Irwin 2017 | | | CT/RT | | 186 | | Post-only | | | | | | Sup AER + RET | | | | | | 12 wks | | | NR | | | | | BW, BMI, LBM %BF, BMD (DXA), 6MWT, insulin, CRP | | | | | |
| Mayo 2014 | | | Mixed | | 26 | | 8, 16, 24 wks | | | | | | HB AER | | | | | | 8 wks | | | Fatigue (FACIT-F, FSI) | | | | | 2MWT | | | | | |
| Schuler 2017 | | | CT | | 70 | | 12, 24 wks | | | | | | Sup AER + RET Sup + HB AER + RET | | | | | | 12 wks | | | Fatigue (MFSI) | | | | | 6MWT, albumin, co-morbidities | | | | | |
| Schwartz 2009 | | | CT | | 101 | | 24, 52 wks | | | | | | HB AER HB RET | | | | | | 52 wks | | | BW, %BF (DXA) | | | | | 12MWT, strength (1-RM) | | | | | |
| ***After treatment*** | | | | | | | | | | | | | | | | | | | | | | | | | | | | | | | | |
| Broderick 2013 | | | CT/RT | | 43 | | 8, 12 wks | | | | | | Sup + HB AER | | | | | | 8 wks | | | Aer capacity (estimated VO_2_peak) | | | | | BW, BMI, WC | | | | | |
| Burnham 2002 | | | S/CT/RT | | 21 | | Post-only | | | | | | Sup AER | | | | | | 10 wks | | | NR | | | | | %BF (SkF), Aer capacity (VO_2_peak), flexibility (sit-and-reach) | | | | | |
| Jones 2014 | | | Mixed | | 90 | | 12, 52 wks | | | | | | Sup + HB AER | | | | | | 12 wks | | | All-cause mortality/ hospitalization (composite) | | | | | Aer capacity (VO_2_peak, RER), 6MWT, HR, all-cause mortality, cardiovascular mortality/hospitalization (composite), cardiovascular mortality/HF hospitalization (composite) | | | | | |
| Kampshoff 2015 | | | CT | | 277 | | Post-only | | | | | | Sup + HB AER + RET (LMI) Sup + HB AER + RET (HI) | | | | | | 12 wks | | | Aer capacity (VO_2_peak, PPO, VT), strength (30s sit-to-stand, HGS), fatigue (MFI) | | | | | BMI, sum of SkF | | | | | |
| Kneis 2019 | | | CT | | 50 | | Post-only | | | | | | Sup AER + Balance (IG) Sup AER (CG) | | | | | | 12 wks | | | Functional balance performance (ST_EO_) | | | | | Aer capacity (VO_2_peak, P_max_, IAT), muscle power (P_max_jump_, jump height), functional balance performance (ST_EC_, MS_EO_ path/duration, MS_Eounstable_ duration), vibration sense (tuning fork) | | | | | |
| Knobf 2017 | | | Non-hormonal therapy | | 154 | | Post-only | | | | | | Sup + HB AER + RET | | | | | | 52 wks | | | BMD (DXA), bone turnover (N-terminal propeptides of type I collagen, osteocalcin) | | | | | BW, BMI, Aer capacity (GXT, HRpeak), HR, BP, HRR, insulin, glucose, HOMA-IR, HbA1-C, total lipid, TG (+), HDL, LDL, TC | | | | | |
| LaStayo 2011 | | | S/CT/RT | | 40 | | Post-only | | | | | | Sup RET | | | | | | 12 wks | | | Strength (isometric force) | | | | | Muscle CSA (MRI), 6MWT, muscle power (stair climb, W), stair descent | | | | | |
| Midtgaard 2013 | | | CT | | 214 | | 24, 52 wks | | | | | | Sup AER + RET | | | | | | 52 wks | | | PA levels (SGPALS), Aer capacity (VO_2_peak) | | | | | Strength (1-RM) | | | | | |
| Pisu 2017 | | | Mixed | | 31 | | Post-only | | | | | | Sup AER (Dance) | | | | | | 12 wks | | | QoL (SF-36) | | | | | 6MWT | | | | | |
| Thorsen 2005 | | | CT | | 111 | | Post-only | | | | | | HB AER + RET | | | | | | 14 wks | | | Aer capacity (estimated VO₂peak) | | | | | None | | | | | |
| Toohey 2016 | | | Mixed | | 16 | | Post-only | | | | | | Sup AER (LVHIIT) Sup AER (CLMIT) | | | | | | 12 wks | | | NR | | | | | BW, HC, WC, LBM, FM, %BF (DXA), 6MWT, strength (5x sit-to-stand), HR, augmentation index, BP, PP, MAP, PWV, CRP, insulin, glucose, full blood count | | | | | |

*Primary outcome when specifically stated.

Tables 2-4 Abbreviations: %BF: body fat %, 1-RM: 1-repetition maximum, 12MWT: 12-min walk test, 6MWT: 6-minute walk test, AAA: aromatic amino acids, AER: aerobic, ALT: alanine aminotransferase, AML: acute myeloid leukemia, AT: anaerobic threshold, BC: body composition, BCAA: branched-chain amino acids, BFI: Brief Fatigue Inventory, BIA: Bioimpedance Analysis, BMD: bone mineral density, BMI: body mass index, BP: blood pressure, BTR: branched-chain amino acid/tyrosine ratio, BW: body weight, CG: control group, CLMIT: continuous low to moderate intensity training, COP: centre of pressure, CRP: C-reactive protein, CRT: chemoradiotherapy, CSA: cross-sectional area, CT: chemotherapy, DE: delayed exercise, DXA: dual-energy X-ray absorptiometry, EE: early exercise, EPO: epoetin alfa, FAB: Fullerton Advanced Balance Scale, FACIT-F: Functional Assessment of Chronic Illness Therapy – Fatigue, FACT-An: Functional Assessment of Cancer Therapy – Anaemia, FACT-C: Functional Assessment of Cancer Therapy – Colorectal, FACT-G: Functional Assessment of Cancer Therapy – General, FFM: fat free mass, FM: fat mass, FMD: flow-mediated dilation, FQL: Fatigue Quality List, FVC: forced vital capacity, GXT: graded exercise test, HADS-D: Hospital Anxiety and Depression Scale, Hb: haemoglobin, HbA1c: glycosylated haemoglobin, HB: home-based, HC: hip circumference, HDL: high-density lipoprotein, HGS: handgrip strength, HI: high intensity exercise, HIG: high-intensity group, HIIE: high-intensity interval exercise, HIIE-T: high-intensity interval exercise-tapered, HOMA-IR: homeostatic model assessment of insulin resistance, HR: heart rate, HRR: heart rate recovery, hs-CRP: high-sensitivity C-reactive protein, IAT: individual anaerobic threshold, ICU: intensive care unit, IG: intervention group, IGF-1: insulin-like growth factor 1, IGFBP-3: IGF binding protein 3, IL6: interleukin 6, IPAQ: International Physical Activity Questionnaire, IVC: inspiratory vital capacity, Lac: blood lactate, LBM: lean body mass, LDL: low-density lipoprotein, LIG: low-intensity group, LMI: low-to-moderate intensity exercise, LVHIIT: low-volume high intensity interval training, MAP: mean arterial pressure, MFI: Multidimensional Fatigue Inventory, MFSI: Multidimensional Fatigue Symptom Inventory. MICE: moderate intensity continuous exercise, MSEO: monopedal stance on stable surface, MSEOunstable: monopedal stance on unstable surface, MVIC: maximum voluntary isometric contraction, MVT: maximal voluntary torque, NR: not reported, NRS: numeric rating scale, PAQ: Physical Activity Questionnaire, PAR: Physical Activity Recall, PF: physical function, PFS: Piper Fatigue Scale, Plts: platelets, Pmax_jump: maximum jump power output, POMS: Profile of Mood States, PPO: peak power output, PROMIS: Patient-Reported Outcomes Measurement Information System, PSQI: Pittsburg Sleep Quality Index, PWV: pulse wave velocity, QLQ-C30: European Organization for Research Treatment Cancer Quality of Life Questionnaire, QLQ-OES18: European Organization for Research Treatment Oesophageal Cancer Quality of Life Questionnaire, QoL: quality of life, RBC: red blood cell count, RER: respiratory exchange ratio, RET: resistance training, ROM: range of motion, RPP: rate pressure product, RQ: respiratory quotient, RT: radiotherapy, S: surgery, sCAM: soluble cell adhesion molecules, SCFS: Schwartz Cancer Fatigue Scale, SCT: stem cell transportation, SGRQ: St. George’s Respiratory Questionnaire, SF-12v2: Short Form 12 item (version) Health Survey, SF-36: 36-Item Short Form Health Survey, SGPALS: Saltin and Grimby physical activity level scale, SkF: skinfolds, SMHB: self-managed home-based, SPADI: Shoulder Pain and Disability Index, SPO2: oxygen saturation, SPPB: short physical performance battery, STEC: semi-tandem stance with eyes closed, STEO: semi-tandem stance with eyes open, sTNFaR2: soluble TNF alpha receptor 2, Sup: supervised, TC: total cholesterol, TG: triglycerides, TUG: timed up and go, VCO2: carbon dioxide production, VE: ventilatory equivalent, VO2: oxygen consumption, VO2peak: peak oxygen consumption, VT: ventilatory threshold, W: watts, WBC: white blood cell count, WC: waist circumference, WHR: waist to hip ratio
